# Opsin transcripts of predatory diving beetles: a comparison of surface and subterranean photic niches

**DOI:** 10.1098/rsos.140386

**Published:** 2015-01-28

**Authors:** Simon M. Tierney, Steven J. B. Cooper, Kathleen M. Saint, Terry Bertozzi, Josephine Hyde, William F. Humphreys, Andrew D. Austin

**Affiliations:** 1 Australian Centre for Evolutionary Biology and Biodiversity and School of Biological Sciences, University of Adelaide, South Australia 5005, Australia; 2 Evolutionary Biology Unit, South Australian Museum, North Terrace, Adelaide, South Australia 5000, Australia; 3 Terrestrial Zoology, Western Australian Museum, Locked Bag 49, Welshpool DC, Western Australia 6986, Australia; 4 School of Animal Biology, University of Western Australia, Nedlands, Western Australia 6907, Australia

**Keywords:** regressive evolution, opsin, pleiotropy, pseudogene, Bidessini, Hydroporini

## Abstract

The regressive evolution of eyes has long intrigued biologists yet the genetic underpinnings remain opaque. A system of discrete aquifers in arid Australia provides a powerful comparative means to explore trait regression at the genomic level. Multiple surface ancestors from two tribes of diving beetles (Dytiscidae) repeatedly invaded these calcrete aquifers and convergently evolved eye-less phenotypes. We use this system to assess transcription of opsin photoreceptor genes among the transcriptomes of two surface and three subterranean dytiscid species and test whether these genes have evolved under neutral predictions. Transcripts for *UV*, *long-wavelength* and *ciliary-type* opsins were identified from the surface beetle transcriptomes. Two subterranean beetles showed parallel loss of all opsin transcription, as expected under ‘neutral’ regressive evolution. The third species *Limbodessus palmulaoides* retained transcription of a *long-wavelength opsin* (*lwop*) orthologue, albeit in an aphotic environment. Tests of selection on *lwop* indicated no significant differences between transcripts derived from surface and subterranean habitats, with strong evidence for purifying selection acting on *L. palmulaoides lwop*. Retention of sequence integrity and the lack of evidence for neutral evolution raise the question of whether we have identified a novel pleiotropic role for *lwop*, or an incipient phase of pseudogene development.

## 2. Introduction

The evolutionary degeneration or loss of traits, known as regressive evolution, has generated considerable discussion in Lamarckian, Darwinian and Neutralist contexts, with eye-loss among subterranean animals being central to the debate (reviewed by [1–8]). Neutral evolutionary theory (non-adaptive evolution) predicts that if genes specifically responsible for vision are not under directional selection, then they should accrue random mutations through genetic drift, ultimately developing into pseudogenes given sufficient time [9–11]. There are very few empirical examples of such genetic loss of function in ‘eye genes’ [12], and instances where visual transduction genes continue to be transcribed in a lightless environment raise the possibility of pleiotropy [13,14]. However, the evolutionary origins of these study systems are rarely of sufficient palaeo-age to allow pseudogenes to develop or exhibit the diversity required for robust comparative phylogenetic assessment.

Tracking the history of an organism as it enters into and diversifies within a novel niche permits the investigation of a ‘natural experiment’ set up by evolution. Such studies are most powerful when contrasting closely related lineages with divergent phenotypes and when there are repeated independent origins of the focal trait [15]. The stygofauna (aquatic obligate subterranean fauna) of arid zone Australia represent a diverse assemblage of subterranean metazoans that derive from surface-water ancestors, which have independently colonized calcrete aquifers during periods of continental aridification in the Plio-Pleistocene [16–20] and so the niche shift may be climate driven. One of the best examples of parallel evolution within this system is that of the predatory diving beetles (Dytiscidae) of the Yilgarn Craton in Western Australia (WA), represented by approximately 100 known stygobitic species from 45 isolated calcrete aquifers [21–23]. A consistent trait among these stygobitic beetles is the complete lack of eyes, lack of pigmentation and wing reduction ([24–26] and references therein). Phylogeographic studies have provided strong evidence that the calcrete aquifers represent closed island systems, with no evidence for gene flow among stygobitic species from different aquifers and an absence of gene flow from surface species over millions of years [19–23,27], potentially long enough for neutral genetic changes to be detected in eye genes that are no longer functional [11]. These attributes, and the independent evolution of the majority of stygobitic beetle species from surface ancestors, provide a powerful model system to explore the long-term genomic changes that accompany regressive evolution of subterranean animals.

The transduction of photons into neural impulses enables animal vision. Visual pigments of photoreceptor cells absorb photons of specific wavelength sensitivities that activate phototransduction cascades. All visual pigments consist of an opsin apo-protein and a chromophore, and the point at which these molecules bind (Schiff-base linkage) determines peak wavelength sensitivity. Point mutations and gene duplication in *opsin* can tune the pigment to alternative wavelength sensitivity, as can rhabdomic filters. The shared ancestry of animal vision [28,29] has made opsin an ideal candidate gene for phylogenetic studies generally, as well as those that track species entry into novel photic niches (e.g. [30,31]). Non-visual opsins are also purported to play regulatory functions in light-mediated processes (e.g. circadian rhythm [32]), and detection of visual opsin expression in arthropod brains may indicate pleiotropic roles (reviewed by [33]).

The aim of this study is to explore the regressive evolution of *opsin* genes from a molecular standpoint. We take a qualitative candidate gene approach and use high-throughput sequencing techniques to compare transcriptomes of two surface and three subterranean dytiscid beetle species that are blind and lack pigmentation. We target opsin phototransduction genes and explore the relative rates of non-synonymous (d*N*) versus synonymous (d*S*) mutations in order to make inferences regarding evolutionary mode. Neutral theory predicts that photoreceptor genes, no longer required in stygobitic taxa, should evolve into pseudogenes. Hence, we should expect an absence of transcription of the ancestral gene (that of surface species) or non-directional rates of mutation (d*N*=d*S*) in genes that continue to be transcribed in an aphotic niche.

## 3. Material and methods

### 3.1 Taxon sampling

Diving water beetles from two tribes of Dytiscidae (Bidessini, Hydroporini) were assessed. Surface species include one bidessine, *Allodessus bistrigatus* (Clark, 1862), and one hydroporine, *Paroster nigroadumbratus* (Clark, 1862); both were collected in September 2012, near Forreston, South Australia (−34.6889°, 138.8933°). Subterranean beetles were collected from bore holes of the Yilgarn region, WA, from which we sourced two bidessine species: *Limbodessus palmulaoides* (Watts & Humphreys, 2006), collected in August 2011, Laverton Downs calcrete, WA (−28.3983°, 122.2038°) and *Neobidessodes gutteridgei* (Watts & Humphreys, 2003), collected in May 2012, Three Rivers Station calcrete, WA (−25.2786°, 119.1834°). Finally, one subterranean hydroporine, *Paroster macrosturtensis* (Watts & Humphreys, 2006), was collected in May 2012, from Sturt Meadows calcrete, WA (−28.7155°, 120.8931°). Specimens were collected under licences SF007802 & SF008440 (W. F. Humphreys), issued by the Department of Environment and Conservation. Each of these species have been the subject of phylogeographic studies [22,34], with further molecular ecological studies for two species (*L. palmulaoides* and *P. macrosturtensis*) [35–37] that have confirmed their long-term independent evolution from sympatric stygobitic species and surface relatives over millions of years. All specimens were preserved in RNAlater.

### 3.2 cDNA synthesis and high-throughput sequencing

Depending on taxon body size [25], 5–10 heads from each species were pooled and homogenized using steel bead disruption. Pooling individuals within a species effectively increases the power of results, particularly when a qualitative presence or absence of expression is desired. Total RNA was purified following standard protocols (RNeasy Plus Micro Kit; Qiagen). Double-stranded cDNA was synthesized and PCR-amplified using the SMARTer cDNA Synthesis Kit and Advantage 2 PCR Kit (Clontech). PCR optimization procedures were gel-verified. cDNA was sequenced on an Illumina HiSeq2000, generating either 100 or 150 bp paired-end reads with TruSeq adapters.

### 3.3 Bioinformatics

#### 3.3.1 Quality control

Raw reads were assessed using FastQC (Babraham Institute) and then edited and trimmed in Cutadapt [38] to filter-out: low-quality ends of reads (phred scores <30), TruSeq barcoded adapters, SMARTer adapters, poly-A tails and sequences less than 25 bp.

#### 3.3.2 Assembly

Transcripts were de novo assembled using the Trinity platform's three-step process, which assembles then aligns contigs via probability derived de Bruijn graphs [39]. Assembled contigs were quality assessed with Bowtie, aligning them back to raw reads to indicate the proportion of proper-paired reads obtained. Read coverage depth for transcripts of interest were ascertained with BEDTools (v. 2.22.0) and summarized in SPSS (v. 21).

#### 3.3.3 Orthologue search

**Figure 1.**
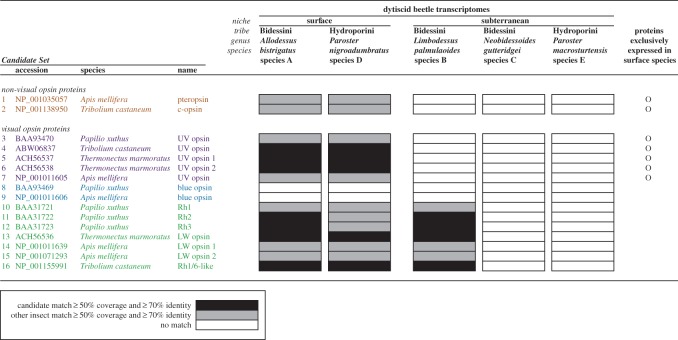
Dytiscid beetle orthologous matches to *Candidate Set* opsin genes. Qualitative representation of positive matches from the five targeted dytiscid beetle transcriptomes, which corresponds to BLAST search results detailed in the electronic supplementary material, table S1—*Candidate Set* gene numbers (1–16) and dytiscid beetle species designations (*a*–*e*). Positive matches (greater than or equal to 50% query coverage and greater than or equal to 70% identity) are defined as: match to target accession (black bar); match to other insect orthologue (grey bar); or no evidence for an orthologous match (white bar). Proteins exclusively expressed by both surface species, but absent from all subterranean species are identified by an open circle.

We used BLASTx to conduct amino acid translated queries of Trinity derived transcripts against a *Candidate Set* of 16 target proteins (listed in figure 1), for non-visual opsins (*ciliary-type* and *pteropsin*) and visual opsins (*UV*, *blue* and *long-wavelength* sensitive), derived from: sunburst diving beetle *Thermonectus marmoratus* (Coleoptera: Dytiscidae); red flour beetle *Tribolium* *castaneum* (Coleoptera: Tenebrionidae); European honeybee *Apis mellifera* (Hymenoptera: Apidae) and Chinese swallowtail butterfly *Papilio xuthus* (Lepidoptera: Papilionidae). Target sequences were obtained from GenBank, accessed June 2013 (figure 1 and electronic supplementary material, tables S1 and S2). We then performed a reciprocal search (tBLASTn), followed by BLASTn searches on the best contig hit (derived from BLASTx) for each candidate protein. An orthologous match was considered positive when: alignment length was greater than or equal to 50%; protein identities were greater than or equal to 30% and nucleotide identities were greater than or equal to 70% [40]. Failing this stringency, BLAST2BLASTn alignments for sequence-pairs were undertaken between best-hit contigs from our study that positively matched an opsin orthologue (subject sequence) and best-hit contigs that failed to match any opsin orthologues on GenBank (query sequence). Best-hit dytiscid transcripts that resulted in a positive orthologue match were deposited in GenBank (accession numbers KP219380–KP219386). A conceptual workflow of these methods is outlined in the electronic supplementary material, figure S1.

#### 3.3.4 Phylogenetics

The *Candidate Set* opsin amino acid sequences (excluding unalignable putative UTR) were first aligned with Clustal Omega [41]; best-hit dytiscid transcripts were manually aligned to the nucleotide translation in Se-Al v. 2.0a11 and reading frames were double checked in MacClade 4.06. MrBayes v. 3.2 was used to construct a 50% majority rule tree, derived from two independent runs of four chains (one cold). We used an objective procedure (least constrained model with ample generation time [42]), to avoid potential errors related to incorrect *a priori* model selection. A GTR + I + *Γ* model was applied to each codon partition, with non-informative priors, unlinked parameters and variable rates permitted. Run length was determined by: (i) likelihood plots, (ii) the average standard deviation of split frequencies, and (iii) estimated sample size. We used a relative burn-in to discard the initial 25% of samples.

#### 3.3.5 Tests of selection

We calculated *P*-distances for amino acid and nucleotide sequences (±s.e). Pairwise Z-tests (Nei–Gojobori method) of neutral evolution, purifying selection and positive selection were undertaken in MEGA 5.2.2, with pairwise deletion of ambiguous sites and 500 bootstrap replicates to estimate variance. We then used HyPhy 2.1 to undertake phylogenetic hypothesis testing using Bayesian inferred trees. We used likelihood ratio tests (LRT) to explore: (i) pairwise relative rates of evolution, (ii) tree-wide global rates of selection versus local rates, (iii) comparison of rates among select branches of the tree, as well as (iv) two site-specific LRT methods that calculate d*N*/d*S* independently at each codon, which differ in analytical robustness: single-likelihood ancestor counts (SLAC) are simplistic; fixed effect likelihood (FEL) methods are more detailed and less susceptible to Type I errors compared with comparable procedures [43].

## 4. Results

### 4.1 Transcriptome assembly

Metrics for the de novo assemblies are summarized in the electronic supplementary material, table S3. We recovered between 50.6 and 78.9 million paired-end reads for each of the five species. Assembled transcripts ranged from 84 667 to 182 240 per species (contig N50 ranged from 565 to 1067 bp) and total aligned reads ranged from 37 to 74.3 million reads.

### 4.2 Searches for orthologous opsin sequences in the dytiscid beetle transcripts

The complete BLAST results are provided in the electronic supplementary material, table S1 and a qualitative representation (presence/absence of orthologous transcript) comparing species is presented in figure 1. Overall, positive matches to 14 of the 16 *Candidate* opsin genes were recovered. Blue-sensitive target candidates were the only opsin class not detected at all. Transcripts from both surface beetle species resulted in positive matches to the two non-visual opsin candidates, *pteropsin* and *c-opsin*, and all five ultraviolet opsin genes (*uvop*). Each of the seven long-wavelength opsin (*lwop*) candidates registered matches in both surface species and also in one subterranean species (*L. palmulaoides*). Transcripts from the remaining two subterranean species, *N. gutteridgei* and *P. macrosturtensis,* did not match any of the 16 *Candidate* opsin genes. Results from surface species are presented independently of subterranean species (below) and correspond to results tabulated in the electronic supplementary material, table S1.

#### 4.2.1 Surface beetle orthologues

Non-visual opsin orthologues were identified among other non-*Candidate* insect taxa (electronic supplementary material, tables S1.1.1, S1.1.3, S1.4.1, S1.4.3). Of these, the best orthologous match was to a *parapinopsin* gene of the silkworm *Bombyx* *mori* (XM_004928326). For the visual opsins, there were amino acid and nucleotide matches to *uvop* of all beetle *Candidates* and the honeybee *Candidate* (electronic supplementary material, tables S1.1.1, S1.1.2, S1.4.1, S1.4.2). The best *uvop* nucleotide matches were to *T. marmoratus*, with *A. bistrigatus* best matched to *T. marmoratus* *uvop-2* (electronic supplementary material, table S1.1.3), and *P. nigroadumbratus* best matched to *T*. *marmoratus* *uvop-1* (electronic supplementary material, table S1.4.3). Both surface dytiscid beetle species returned amino acid matches to blue opsin *Candidates*; however, nucleotide searches of these best-hit transcripts did not match to any blue opsin in GenBank, and the best matches were to *uvop* of *T. marmoratus*. Long-wavelength opsin *Candidates* yielded the highest scores of all our BLAST orthologue searches. Both focal surface dytiscid beetle species transcripts returned the highest scoring nucleotide matches (BLASTn, electronic supplementary material, tables S1.1.2, S1.4.2), which were most closely affiliated to *lwop* of the North American dytiscid beetle *T. marmoratus*.

#### 4.2.2 Subterranean beetle orthologues

A single transcript from the *L. palmulaoides* assembly rated as the best hit for all opsin *Candidates* in the amino acid search (electronic supplementary material, table S1.2.1) and the best nucleotide match to this transcript was to *T. marmoratus* *lwop* (electronic supplementary material, table S1.2.3). No positive matches to any of the opsin *Candidates* were found for the two subterranean species *N. gutteridgei* and *P. macrosturtensis*. However, for these two species, the majority of GenBank nucleotide matches (BLASTn derived) to best-hit transcripts (derived from BLASTx) were to other members of the G protein-coupled receptor family (which includes opsins) that were not related to phototransduction (electronic supplementary material, tables S1.3.3, S1.5.5.3). This suggests that our assemblies were of high quality and capable of discerning transcripts at high fidelity.

### 4.3 Orthologue coverage depth

**Figure 2.**
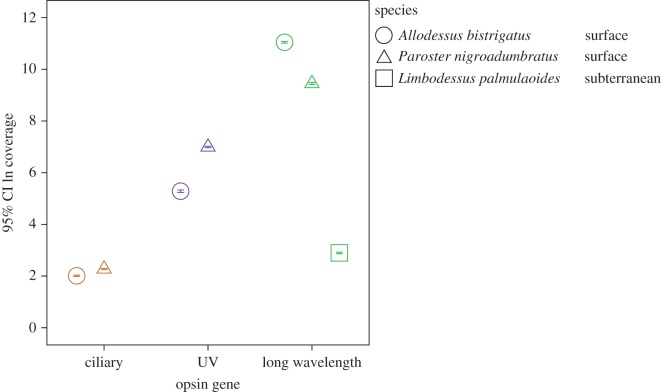
Relative sequence coverage depth for identified opsin orthologues. Mean sequence coverage depth is plotted on a lognormal scale. The relative coverage is indicated for each of the seven indentified opsin orthologues for surface (*A. bistrigatus*, circle; *P. niogroadumbratus*, triangle) and subterranean (*L. palmulaoides*, square) species. Opsin copy is identified by colour: ciliary-type (brown), UV (violet) and long wavelength (green).

Depth of sequencing coverage for each of the seven identified opsin orthologues (un-edited) is summarized in table 1 and plotted on a lognormal scale in figure 2. The non-visual *c-opsin* from surface species exhibited the lowest levels of mean coverage per nucleotide base (less than 12 times coverage). The visual opsins of surface beetle species showed coverage at orders of magnitude higher (*uvop* means less than 1220 times coverage; *lwop* means less than 69 015 times coverage). The *lwop* of the subterranean *L. palmulaoides* displayed a mean sequence coverage depth of 22.51 (±s.e. 0.57) per nucleotide base, which is double the level of coverage found for *c-opsin* in the two surface species; this suggests that our finding of the expression of a visual opsin in a blind subterranean beetle is not an aberration.

**Table 1.** Dytiscid opsin sequence coverage depth. Mean sequence coverage depth per nucleotide base (±s.e.) for transcripts of dytiscid beetle opsin orthologues. The maximum depth for a single nucleotide base within each transcript is presented in square brackets and the sample size (*n*) represents the full length of the unedited transcript (number of bases). The subterranean species is shaded grey.

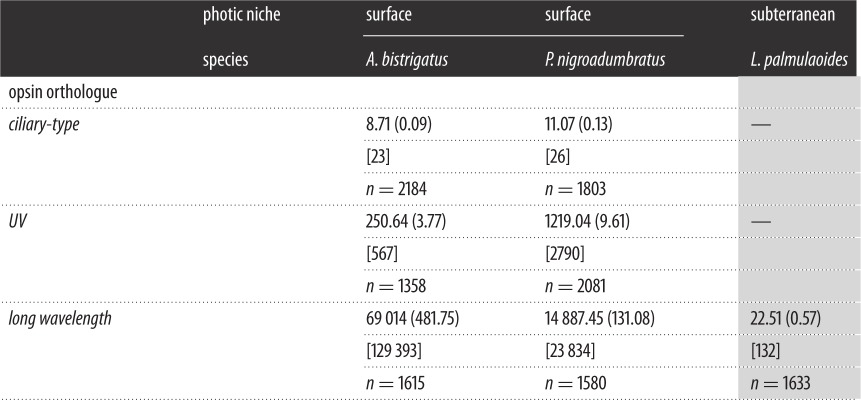

### 4.4 Phylogenetic assessment of predicted opsin transcripts

**Figure 3.**
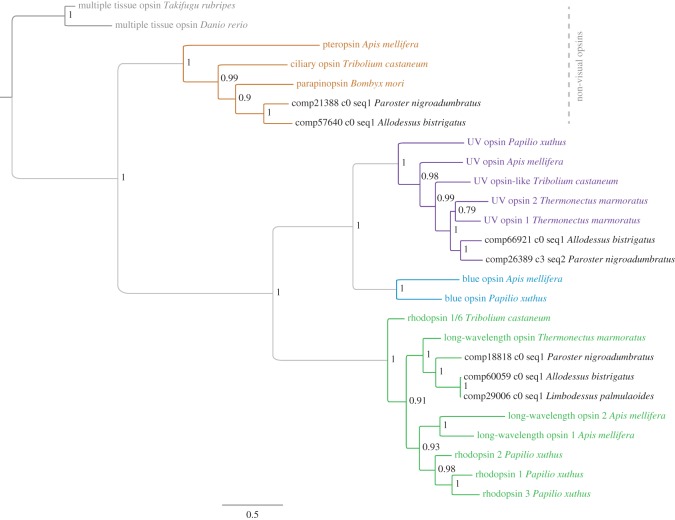
Opsin phylogeny. Majority rule consensus tree of visual and non-visual opsin photoreceptors derived from Bayesian inference, with posterior probability node support. Tree branches and terminal taxa are colour coded to identify *Candidate Set* opsin gene class from figure 1: ciliary (brown), UV (violet), blue (blue), long wavelength (green) and outgroups (dark grey).

All best-hit transcripts that returned positive matches to opsins in the *Candidate Set*, and detailed in the BLAST results above, were then placed within a comparative phylogenetic framework. We augmented the 16 candidate opsin matrix with an additional three non-visual opsins (figure 3; electronic supplementary material, table S2). Based on the aforementioned BLAST results, we included the *parapinopsin* of *B. mori*. To root the tree, we included two vertebrate non-visual opsins as outgroups: *multiple tissue opsin* of *Takifugu* *rubripes* and *teleost multiple tissue opsin* of *Danio rerio*. An aligned nucleotide matrix comprising the *Candidate Set* opsins and their dytiscid beetle best-hit positive match transcripts (26 sequences, 1005 nucleotides [44]) was subjected to a Bayesian phylogenetic analysis of 10 million iterations.

The resultant Bayesian tree (with posterior probability node support) corroborates BLAST search results and is presented in figure 3. All opsin classes (ciliary, UV, blue and long-wavelength) formed fully resolved monophyletic groups with maximal posterior probability support. The BLAST-predicted non-visual opsin of *P. nigroadumbratus* was grouped with the parapinopsin-like *B. mori*, nested within the invertebrate ciliary opsin clade, which formed a sister group to the remaining rhabdomeric visual opsins. The predicted *uvop* transcripts of both focal surface dytiscid species (*A. bistrigatus* and *P. nigroadumbratus*) formed a distal monophyletic group that was most closely related to UV opsins of *T. marmoratus*, the only other dytiscid beetle in the matrix. For long-wavelength opsin the same family level grouping for Dytiscidae was apparent. The only subterranean dystiscid to express an opsin (*L. palmulaoides*) formed a monophyletic group with the predicted *lwop* transcript of the other tribal member of Bidessini, *A. bistrigatus*. The *lwop* transcript of *P. nigroadumbratus* (Hydroporini) formed the sister branch to the Bidessini clade and *T. marmoratus* (Aciliini) was the next basal *lwop* branch. The dytiscid long-wavelength opsin clade was a sister group to bee and butterfly clades; however, the *T. castaneum* copy was recovered as sister lineages to all other long-wavelength opsins.

### 4.5 Comparative tests of evolutionary rates between surface and subterranean beetles

Considering that a blind (eye-less) species in an aphotic niche has expressed a visual opsin, we undertook analyses of evolutionary rates on *lwop*. It is recommended that codon based likelihood estimates of d*N*/d*S* are best undertaken on single gene products [45], so we removed all non-*lwop* opsin copies from their respective matrices for comparative analyses. Pairwise analyses were only performed on beetles in order to contrast surface and subterranean lineages. For phylogenetically informed analyses, the number and phylogenetic distance of taxa within an alignment matrix can influence results, hence we ran two datasets: one containing dytiscid beetles only (*n*=4), and a second matrix with a wider variety of insect outgroups including the red flour beetle, two bee and three butterfly *lwop* copies (*n*=10).

#### 4.5.1 Pairwise estimates of d*N*/d*S*

Table 2 displays pairwise results that compare the stygobitic *L. palmulaoides* with all other surface dytiscids, followed by comparisons among the surface taxa. All *P*-distance comparisons of *lwop* rejected neutral evolution, showed strong support for purifying selection and showed no evidence of positive selection. *Lwop* sequences of the two Bidessini species were very similar, with a nucleotide *P*-distance of only 0.006, and amino acid products were identical.

**Table 2.** Pairwise assessment of dytiscid beetle *long-wavelength opsin. P*-distances calculated using amino acid (aa) and nucleotide (nuc) alignments. Hypothesis-based assessment (*Z*-tests) of neutral evolution as the null state (H_0_), whereby non-synonymous substitutions are equal to synonymous substitutions (d*N*=d*S*), tested against the following alternative hypotheses (H_a_):H_a_ 1—non-neutral evolution d*N*≠d*S*; H_a_ 2—purifying selection d*N*<d*S*; and H_a_ 3—positive selection d*N*>d*S*. Grey shading indicates an aphotic niche.

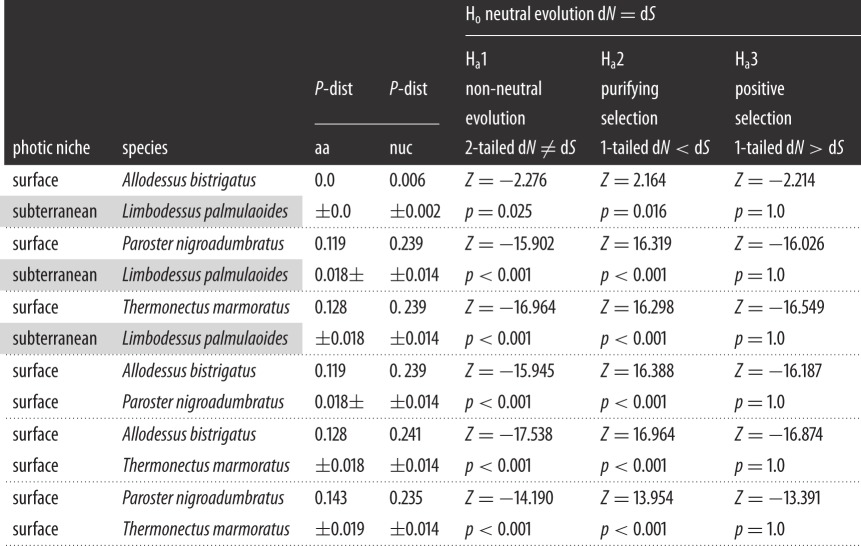

#### 4.5.2 Phylogenetically informed likelihood estimates of d*N*/d*S*

For the beetle-only matrix, AIC model tests selected 010123 for the nucleotide model and F81 (000000) for the codon model. The global d*N*/d*S* ratio (total evidence across the entire matrix and tree) was *ω*=0.0239475; however, an LRT comparing global *ω*(H_o_) with local *ω*(H_1_) rates favoured the alternate hypothesis (χ_4_^2^ = 2660.59, *p*<0.001). The expanded insect dataset (*n*=10) was analysed with AIC determined nucleotide (012032) and codon (010110) models. There was no recombination in the alignment and the global d*N*/d*S* ratio *ω*=0.0463382 was rejected in favour of local rates (χ_16_^2^ = 36.0746, *p*=0.003). Based on these rejections of the shared global *ω* ratio, we then explored a range of analyses contrasting branches within the tree.

#### 4.5.3 Variation in d*N*/d*S* among branches

We compared evolutionary rates among various terminal branches of the tree using LRTs, exploring different local rate variations: (i) no site-to-site variation, (ii) d*N* rate variation only and (iii) independent d*N* and d*S* rate variation. We tested three alternative sets of branches against the rest of the tree: we first compared the subterranean *L. palmulaoides* terminal branches (*n*=1), then the surface beetle terminal branches (*n*=3), and finally all terminal branches against all internal branches (*n*=4). None of these comparisons showed significantly different rates of variation among select branches in the tree when tested against a global rate (electronic supplementary material, table S4). Similarly, the expanded insect dataset did not result in any significant LRT comparisons (electronic supplementary material, table S4).

#### 4.5.4 Variation in d*N*/d*S* at the site-specific (codon) level

Site-specific methods reiterated the results from pairwise Z-tests. A minimum of 10 sequences is recommended for these analyses [43], thus we only present results from the expanded ‘insect’ dataset. The *lwop* matrix contained 328 codons, for which there were 498 distinct sites. Approximately one-half to two-thirds were estimated as undergoing purifying selection, depending on the analytical procedure: SLACs identified 153 codons (47%); fixed effects likelihood analysis identified 214 codons (65%). We also used FEL to compare the dytiscid beetle sub-tree in isolation to the remainder of the branches in the tree, which recovered 147 codons (45%), followed by the surface (*A. bistrigatus*) and subterranean (*L. palmulaoides*) bidessine species in isolation, which yielded only three codons (less than 1%). None of these perturbations identified any codons as undergoing positive selection. Thus, the subterranean and surface bidessines indicate very minimal change in the long-wavelength opsin expressed in the aphotic environment.

## 5. Discussion

Our initial aim was to qualitatively identify transcribed opsin genes in organisms occupying radically different photic niches. Under a neutral evolution theory, we expected detection of opsin transcripts in surface lineages, but a lack of transcription in subterranean lineages. Such data then provide grounds for subsequent comparative exploration of genomic DNA to assess whether non-adaptive (neutral) evolution has rendered these genes non-functional and contributed to the regressive evolution of eyes in stygofauna.

Our results show that in three independently evolved subterranean dytiscid beetle species nearly all opsin photoreceptor genes lacked evidence of transcription (except *lwop* in *L. palmulaoides*), whereas the two surface-water species showed evidence of expression for a full suite of insect visual and non-visual opsins (excepting blue opsin). This result is suggestive of parallel molecular evolution of a loss of photoreceptor function, more specifically the ‘neutral’ regressive evolution of eye genes. An alternative explanation for a lack of opsin gene transcription in the subterranean species is that opsins were transcribed, but at extremely low levels that were insufficient to allow detection (i.e. a problem of read coverage). However, transcriptome sequence reads of the subterranean species were as high (or higher) than that of the surface species (electronic supplementary material, table S3), and greater than that used in other similar studies of subterranean taxa that identified transcription of opsin genes [46].

The *lwop* orthologue transcribed by the subterranean dytiscid *L. palmulaoides* did not exhibit rates of mutation consistent with neutral evolution (d*N*=d*S*); rather, there was strong evidence for purifying selection (d*N*<d*S*). The translated opsin product seems to be structurally functional as it is almost identical in sequence to orthologues from surface species. A similar result was found for the cave-dwelling carrion beetle *Ptomaphagus* *hirtus* (which possesses a residual eye lens), for which transcriptome evidence suggests a reduced, but apparently functional, visual system [46].

Opsin apo-proteins bind with chromophores to form photopigment molecules that are responsible for the transduction of photons of light into neurophysiological signals, and there are visual and non-visual opsins. Rhabdomeric opsins of diurnally active insect eyes are typically sensitive to UV, blue and long wavelengths, which enables tri-chromatic colour vision; a number of non-visual ciliary opsins have been identified, but their function remains enigmatic. Our study has identified two visual opsins in surface-water dytiscid beetles orthologous to insect UV and long-wavelength classes and one non-visual ciliary opsin.

We did not identify a blue opsin orthologue at all in dytiscids, but note that its loss has been documented for the red flour beetle [47], and the only other publicly available sequence data for a dytiscid beetle's opsins is from larvae of the sunburst diving beetle, which also lacks blue sensitivity [48,49]. Maintenance of blue sensitivity is likely to be dependent on the photic niche of the focal species, and electroretinogram studies suggest day-active predatory lady beetles do possess blue-sensitive (420 nm) photopigments [50].

The non-visual opsin we identified in surface-water dytiscids shows similarity to honeybee *pteropsin* [51] and most closely resembles a predicted *parapinopsin*-like gene from silk worms; *parapinopsin* is a non-visual opsin expressed in the pineal and parietal organs of vertebrates [52,53]. However, the role of non-visual ciliary-type opsins in insects is not comprehensively understood.

### 5.1 Opsin transcription in an aphotic subterranean habitat: pleiotropy or incipient pseudogene?

Our finding of expression of the visual *lwop* by the subterranean *L. palmulaoides* is counterintuitive. As indicated in the opsin phylogeny branch lengths and *P*-distances, there is minimal sequence difference between *lwop* from *A. bistrigatus* and *L. palmulaoides*, there is no evidence for neutral evolution, there are no aberrant stop codons indicative of pseudogenes and evolutionary rates appear to be comparable to both close and distant phylogenetic insect relatives. So are we observing a precursory phase of pseudogene development? This would seem plausible if aphotic lineages have only very recently diverged from photic (surface) ancestors, as in some cavefish [12]. However, the subterranean beetles are proposed to have colonized calcrete aquifers during the Plio-Pleistocene aridification of Australia [22], with a major radiation of these subterranean beetles estimated between 3 and 8 Ma, based on uncalibrated molecular-clock analyses [23].

While the transcription of non-functional opsins over considerable evolutionary periods is possible (e.g. more than 100 Ma [54]), there are other examples whereby cave-adapted animals maintain seemingly functional visual opsins. Arthropod studies comparing cave- and surface-dwelling crayfish species [13] and amphipod populations [14] imply that there has been no loss of opsin function in the aphotic environment. Both studies conclude that this constitutes evidence for a pleiotropic role for the respective visual opsin proteins, but supporting empirical evidence for pleiotropy is limited. It should be noted that: (i) cave crayfish are cephalically blind but possess non-visual *caudal* photoreceptors [55] that may be the primary means of photoreception [56], whereas the cave amphipods possess reduced eyes with far fewer ommatidia [14,57], p. 111); and (ii) neither of these aforementioned cave environments physically isolate the organisms from their respective surface relatives, which is the case for the calcrete aquifers that house the Australian dytiscid beetle stygofauna [17,35,36], and an important distinction between our study system and most other explorations of regressive evolution in eye genes.

Among insects there is evidence of functional visual opsin expression in the larval and adult brains of butterflies and bees: silkworm *lwop* [58], hawkmoth *lwop* [59] and bumblebee *uvop* [33]. Lampel *et al.* [59] speculated on the circadian role of invertebrate non-visual opsins, and in at least one vertebrate, blind cavefish, point mutations (stop codons) in both *melanopsin* and *teleost multiple tissue opsin* are implicated in the de-activation of diurnal circadian rhythm [32]. The retention (‘recycling’) of larval-stage visual organs in adults of some endopterygote insects are also implicated in deep-brain circadian entrainment (notably *Drosophila, Tribolium* and *Thermonectus*—reviewed in [60,61]), suggesting ontogenesis of these insect visual systems is complex and deserving of considerable attention.

The expression of opsin in the early developmental phases of *Astyanax* cave-dwelling fish with degenerate eyes has been used as evidence for a pleiotropic role [62]. Juvenile cavefish continue to express an apparently functional red-opsin protein (cf. surface lineages [63]) for the first 3–6 days of development, which would be prior to the emergence of a functional eye in surface lineages. Langecker *et al.* [62] postulated that opsin might play a structurally important role in the morphogenesis of normal eye development, but argued that eye regression must, therefore, be due to malfunctions in regulatory genes in *Astyanax* cavefish.

More recent evolutionary studies on an alternative cavefish system, the amblyopsids [12], suggest that retention of a structurally sound opsin in blind morphs is a function of evolutionary time relative to colonization of the aphotic niche: amblyopsid species that have persisted for considerably longer periods in caves (up to 10 Ma) show evidence of a loss of selective constraint on opsins and subsequent pseudogene development [12]. We do not currently have a wide enough sampling of taxa in our system to be confident in linking the comparative age of subterranean colonization by *L. palmulaoides*, relative to other stygobitic species, as a factor contributing to the retention of a seemingly functional *lwop* protein.

## 6. Conclusion

This study presents a comparative assessment of the expression of visual and non-visual opsin proteins in surface (photic) and subterranean (aphotic) habitats of dytiscid water beetles. The blind subterranean species *L. palmulaoides* expressed a structurally functional copy of *lwop* that is indistinguishable in amino acid sequence from its surface relative *A. bistrigatus*, and these affinities are supported by fully resolved phylogenetic trees. We cannot rule out a pleiotropic function for *lwop* in dytiscid beetles, but note that an incipient phase of pseudogene development is also a plausible explanation for our finding. In-solution hybridization capture methods, which use RNA-baits to target nuclear DNA, carried out on a much wider sampling of species from this system will permit a more considered assessment of potential pseudogene development among subterranean species. This project embodies the power of genomic-level investigations of evolutionary ecology, whereby the effects of environment and genetic inheritance can be dovetailed to explore phenotypic development and the associated physiology via gene transcription. Our finding of a general lack of opsin expression among three independently evolved subterranean diving beetle lineages suggests a parallel loss of opsin expression in an aphotic environment, which we argue is indicative of ‘neutral’ regressive evolution.

## Supplementary Material

ESM for opsins of surface & subterranean diving beetles

## Data accessibility

Sequences of opsin orthologues are deposited in GenBank under accession numbers KP219380–KP219386, and nexus file alignment data are available from the Dryad Digital Repository: http://dx.doi.org/10.5061/dryad.0dq8s.

## References

[1] Dobzhansky T 1970 Genetics of the evolutionary process. New York, NY: Columbia University Press.

[2] Culver DC, Wilkens H 2000 Critical review of the relevant theories of the evolution of subterranean animals. In Ecosystems of the world 30: Subterranean Ecosystems (eds Wilkens H, Culver DC, Humphreys WF), pp. 381–398. Amsterdam, The Netherlands: Elsevier Science BV.

[3] Porter ML, Crandall KA 2003 Lost along the way: the significance of evolution in reverse. Trends Ecol. Evol. 18, 541–547. (doi:10.1016/S0169-5347(03)00244-1)

[4] Jeffery WR 2009 Regressive evolution in Astyanax cavefish. Annu. Rev. Genet. 43, 25–47. (doi:10.1146/annurev-genet-102108-134216) 19640230 10.1146/annurev-genet-102108-134216 PMC3594788

[5] Romero A 2009 Cave biology: life in darkness. Cambridge, UK: Cambridge University Press.

[6] Wilkens H 2010 Genes, modules and the evolution of cave fish. Heredity 105, 413–422. (doi:10.1038/hdy.2009.184) 20068586 10.1038/hdy.2009.184

[7] Friedrich M 2013 Biological clocks and visual systems in cave-adapted animals at the dawn of speleogenomics. Integr. Comp. Biol. 53, 50–67. (doi:10.1093/icb/ict058) 23720528 10.1093/icb/ict058

[8] Rétaux S, Casane D 2013 Evolution of eye development in the darkness of caves: adaptation, drift, or both? EvoDevo 4, 26 (doi:10.1186/2041-9139-4-26) 24079393 10.1186/2041-9139-4-26 PMC3849642

[9] Kimura M 1968 Evolutionary rate at the molecular level. Nature 217, 624–626. (doi:10.1038/217624a0) 5637732 10.1038/217624a0

[10] Yokoyama S, Meany A, Wilkens H, Yokoyama R 1995 Initial mutational steps towards loss of opsin gene function in cavefish. Mol. Biol. Evol. 12, 527–532 7659009 10.1093/oxfordjournals.molbev.a040233

[11] Leys R, Cooper SJB, Strecker U, Wilkens H 2005 Regressive evolution of an eye pigment gene in independently evolved eyeless subterranean diving beetles. Biol. Lett. 1, 496–499. (doi:10.1098/rsbl.2005.0358) 17148242 10.1098/rsbl.2005.0358 PMC1626372

[12] Niemiller ML, Fitzpatrick BM, Shah P, Schmitz L, Near TJ 2013 Evidence for repeated loss of selective constraint in rhodopsin of amblyopsid cavefishes (Teleostei: Amblyopsidae). Evolution 67, 732–748. (doi:10.1111/j.1558-5646.2012.01822.x) 23461324 10.1111/j.1558-5646.2012.01822.x

[13] Crandall KA, Hillis DM 1997 Rhodopsin evolution in the dark. Nature 387, 667–668. (doi:10.1038/42628) 9192889 10.1038/42628

[14] Carlini DB, Satish S, Fong DW 2013 Parallel reduction in expression, but no loss of functional constraint, in two opsin paralogs within cave populations of *Gammarus minus* (Crustacea: Amphipoda). BMC Evol. Biol. 13, 89 (doi:10.1186/1471-2148-13-89) 23617561 10.1186/1471-2148-13-89 PMC3651389

[15] Harvey PH, Pagel MD 1991 The comparative method in evolutionary biology. Oxford, UK: Oxford University Press.

[16] Morgan KH 1993 Development, sedimentation and economic potential of palaeoriver systems of the Yilgarn Craton of Western Australia. Sediment. Geol. 85, 637–656. (doi:10.1016/0037-0738(93)90106-F)

[17] Humphreys WF 2012 Diversity patterns in Australia. In Encyclopedia of caves, 2nd edn (eds White WB, Culver DC), pp. 203–219. Oxford,UK: Academic Press.

[18] Cooper SJB, Bradbury JH, Saint KM, Leys R, Austin AD, Humphreys WF 2007 Subterranean archipelago in the Australian arid zone: mitochondrial DNA phylogeography of amphipods from central Western Australia. Mol. Ecol. 16, 1533–1544. (doi:10.1111/j.1365-294X.2007.03261.x) 17391274 10.1111/j.1365-294X.2007.03261.x

[19] Cooper SJB, Saint KM, Taiti S, Austin AD, Humphreys WF 2008 Subterranean archipelago: mitochondrial DNA phylogeography of stygobitic isopods (Oniscidea: *Haloniscus*) from the Yilgarn region of Western Australia. Invertebr. Syst. 22, 195–203. (doi:10.1071/IS07039)

[20] Juan C, Guzik MT, Jaume D, Cooper SJB 2010 Evolution in caves: Darwin's ‘wrecks of ancient life’ in the molecular era. Mol. Ecol. 19, 3865–3880. (doi:10.1111/j.1365-294X.2010.04759.x) 20637049 10.1111/j.1365-294X.2010.04759.x

[21] Cooper SJB, Hinze S, Leys R, Watts CHS, Humphreys WF 2002 Islands under the desert: molecular systematics and evolutionary origins of stygobitic water beetles (Coleoptera: Dytiscidae) from central Western Australia. Invertebr. Syst. 16, 589–598. (doi:10.1071/IT01039)

[22] Leys R, Watts CHS, Cooper SJB, Humphreys WF 2003 Evolution of subterranean diving beetles (Coleoptera: Dytiscidae: Hydroporini, Bidessini) in the arid zone of Australia. Evolution 57, 2819–2834. (doi:10.1111/j.0014-3820.2003.tb01523.x) 14761060 10.1111/j.0014-3820.2003.tb01523.x

[23] Leijs R, van Nes EH, Watts CH, Cooper SJB, Humphreys WF, Hogendoorn K 2012 Evolution of blind beetles in isolated aquifers: a test of alternative modes of speciation. PLoS ONE 7, 34260 (doi:10.1371/journal.pone.0034260) 10.1371/journal.pone.0034260 PMC3316697 22479581

[24] Watts CHS, Humphreys WF 2004 Thirteen new Dytiscidae (Coleoptera) of the genera *Boongurrus* Larson, *Tjirtudessus* Watts & Humphreys and *Nirripirti* Watts & Humphreys, from underground waters in Australia. Trans. R. Soc. South Aust. 128, 99–129

[25] Watts CHS, Humphreys WF 2006 Twenty-six new Dytiscidae (Coleoptera) of the genera *Limbodessus* Guignot and *Nirripirti* Watts & Humphreys, from underground waters in Australia. Trans. R. Soc. South Aust. 130, 123–185

[26] Watts CHS, Humphreys WF 2009 Fourteen new Dytiscidae (Coleoptera) of the genera *Limbodessus* Guignot, *Paroster* Sharp, and *Exocelina* Broun from underground waters in Australia. Trans. R. Soc. South Aust. 133, 62–107

[27] Guzik MT, Abrams KM, Cooper SJB, Humphreys WF, Cho J-L, Austin AD 2008 Phylogeography of the ancient Parabathynellidae (Crustacea: Bathynellacea) from the Yilgarn region of Western Australia. Invertebr. Syst. 22, 205–216. (doi:10.1071/IS07040)

[28] Plachetzki DC, Fong CR, Oakley TH 2010 The evolution of phototransduction from an ancestral cyclic nucleotide gated pathway. Proc. R. Soc. B 277, 1963–1969. (doi:10.1098/rspb.2009.1797) 10.1098/rspb.2009.1797 PMC2880087 20219739

[29] Porter ML, Blasic JR, Bok MJ, Cameron EG, Pringle T, Cronin TW, Robinson PR 2012 Shedding new light on opsin evolution. Proc. R. Soc. B 279, 3–14. (doi:10.1098/rspb.2011.1819) 10.1098/rspb.2011.1819 PMC3223661 22012981

[30] Yokoyama S 2008 Evolution of dim-light and color vision pigments. Annu. Rev. Genomics Hum. Genet. 9, 259–282. (doi:10.1146/annurev.genom.9.081307.164228) 18544031 10.1146/annurev.genom.9.081307.164228

[31] Tierney SM, Sanjur O, Grajales GG, Santos LM, Bermingham E, Wcislo WT 2012 Photic niche invasions: phylogenetic history of the dim-light foraging augochlorine bees. Proc. R. Soc. B 279, 794–803. (doi:10.1098/rspb.2011.1355) 10.1098/rspb.2011.1355 PMC3248740 21795273

[32] Cavallari N 2011 A blind circadian clock in cavefish reveals that opsins mediate peripheral clock photoreception. PLoS Biol. 9, 1001142 (doi:10.1371/journal.pbio.1001142) 10.1371/journal.pbio.1001142 PMC3167789 21909239

[33] Spaethe J, Briscoe AD 2005 Molecular characterization and expression of the UV opsin in bumblebees: three ommatidial subtypes in the retina and a new photoreceptor organ in the lamina. J. Exp. Biol. 208, 2347–2361. (doi:10.1242/jeb.01634) 15939775 10.1242/jeb.01634

[34] Leys R, Watts CHS 2008 Systematics and evolution of the Australian subterranean hydroporine diving beetles (Dytiscidae), with notes on *Carabhydrus*. Invertebr. Syst. 22, 217–225. (doi:10.1071/IS07034)

[35] Guzik MT, Cooper SJB, Humphreys WF, Austin AD 2009 Fine-scale comparative phylogeography of a sympatric sister species triplet of subterranean diving beetles from a single calcrete aquifer in Western Australia. Mol. Ecol. 18, 3683–3698. (doi:10.1111/j.1365-294X.2009.04296.x) 19674311 10.1111/j.1365-294X.2009.04296.x

[36] Guzik MT, Cooper SJB, Humphreys WF, Ong S, Kawakami T, Austin AD 2011 Evidence for population fragmentation within a subterranean aquatic habitat in the Western Australian desert. Heredity 107, 215–230. (doi:10.1038/hdy.2011.6) 21343944 10.1038/hdy.2011.6 PMC3183951

[37] Bradford TM, Humphreys WF, Austin AD, Cooper SJB 2014 Identification of trophic niches of subterranean diving beetles in a calcrete aquifer by DNA and stable isotope analyses. Mar. Freshwater Res. 65, 95–104. (doi:10.1071/MF12356)

[38] Martin M 2011 Cutadapt removes adapter sequences from high-throughput sequencing reads. EMBnet J. 17, 10–12. (doi:10.14806/ej.17.1.200)

[39] Haas BJ 2013 *De novo* transcript sequence reconstruction from RNA-seq using the Trinity platform for reference generation and analysis. Nat. Protoc. 8, 1494–1512. (doi:10.1038/nprot.2013.084) 23845962 10.1038/nprot.2013.084 PMC3875132

[40] Tommaso PD, Moretti S, Xenarios I, Orobitg M, Montanyola A, Chang J-M, Taly J-F, Notredame C 2011 T-Coffee: a web server for the multiple sequence alignment of protein and RNA sequences using structural information and homology extension. Nucleic Acids Res. 39, 13 (doi:10.1093/nar/gkr245) 10.1093/nar/gkr245 PMC3125728 21558174

[41] Sievers F 2011 Fast, scalable generation of high-quality protein multiple sequence alignments using Clustal Omega. Mol. Syst. Biol. 7, 539 (doi:10.1038/msb.2011.75) 21988835 10.1038/msb.2011.75 PMC3261699

[42] Berger J 2006 The case for objective Bayesian analysis. Bayesian Anal. 1, 385–402. (doi:10.1214/06-BA115)

[43] Kosakovsky Pond SL, Frost SDW 2005 Not so different after all: a comparison of methods for detecting amino acid sites under selection. Mol. Biol. Evol. 22, 1208–1222. (doi:10.1093/molbev/msi105) 15703242 10.1093/molbev/msi105

[44] Tierney SM, Cooper SJB, Saint KM, Bertozzi T, Hyde J, Humphreys WF, Austin AD 2015 Data from: opsin transcripts of predatory diving beetles: a comparison of surface and subterranean photic niches. Dryad Digital Repository. (doi:10.5061/dryad.0dq8s) 10.1098/rsos.140386 PMC4448788 26064586

[45] Delport W, Poon AF, Frost SDW, Kosakovsky Pond SL 2010 Datamonkey 2010: a suite of phylogenetic analysis tools for evolutionary biology. Bioinformatics 26, 2455–2457. (doi:10.1093/bioinformatics/btq429) 20671151 10.1093/bioinformatics/btq429 PMC2944195

[46] Friedrich M, Chen R, Daines B, Bao R, Caravas J, Rai PK, Zagmajster M, Peck SB 2011 Phototransduction and clock gene expression in the troglobiont beetle *Ptomaphagus hirtus* of Mammoth cave. J. Exp. Biol. 214, 3532–3541. (doi:10.1242/jeb.060368) 21993781 10.1242/jeb.060368

[47] Jackowska M, Bao R, Liu Z, McDonald EC, Cook TA, Friedrich M 2007 Genomic and gene regulatory signatures of cryptozoic adaptation: loss of blue sensitive photoreceptors through expansion of long wavelength-opsin expression in the red flour beetle *Tribolium castaneum*. Front. Zool. 4, 24 (doi:10.1186/1742-9994-4-24) 18154648 10.1186/1742-9994-4-24 PMC2254409

[48] Maksimovic S, Cook TA, Buschbeck EK 2009 Spatial distribution of opsin-encoding mRNAs in the tiered larval retinas of the sunburst diving beetle *Thermonectus marmoratus* (Coleoptera: Dytiscidae). J. Exp. Biol. 212, 3781–3794. (doi:10.1242/jeb.031773) 19915119 10.1242/jeb.031773 PMC2778735

[49] Maksimovic S, Layne JE, Buschbeck EK 2011 Spectral sensitivity of the principal eyes of sunburst diving beetle, *Thermonectus marmoratus* (Coleoptera: Dytiscidae), larvae. J. Exp. Biol. 214, 3524–3531. (doi:10.1242/jeb.058990) 21993780 10.1242/jeb.058990

[50] Lin J-T 1993 Identification of photoreceptor locations in the compound eye of *Coccinella septempunctata* Linnaeus (Coleoptera, Coccinellidae). J. Insect Physiol. 39, 555–562. (doi:10.1016/0022-1910(93)90037-R)

[51] Velarde RA, Sauer CD, Walden KKO, Fahrbach SE, Robertson HM 2005 Pteropsin: a vertebrate-like non-visual opsin expressed in the honey bee brain. Insect Biochem. Mol. Biol. 35, 1367–1377. (doi:10.1016/j.ibmb.2005.09.001) 16291092 10.1016/j.ibmb.2005.09.001

[52] Blackshaw S, Snyder SH 1997 Parapinopsin, a novel catfish opsin localized to the parapineal organ, defines a new gene family. J. Neurosci. 17, 8083–8092 9334384 10.1523/JNEUROSCI.17-21-08083.1997 PMC6573767

[53] Wada S, Kawano-Yamashita E, Koyanagi M, Terakita A 2012 Expression of UV-sensitive parapinopsin in the iguana parietal eyes and its implication in UV-sensitivity in vertebrate pineal-related organs. PLoS ONE 7, 39003 (doi:10.1371/journal.pone.0039003) 10.1371/journal.pone.0039003 PMC3375259 22720013

[54] Yokoyama S, Starmer WT, Lui Y, Tada T, Britt L 2014 Extraordinarily low evolutionary rates of short wavelength-sensitive opsin pseudogenes. Gene 534, 93–99. (doi:10.1016/j.gene.2013.09.114) 24125953 10.1016/j.gene.2013.09.114 PMC3852691

[55] Larimer J 1966 A functional caudal photoreceptor in blind cavernicolous crayfish. Nature 210, 204–205. (doi:10.1038/210204b0) 5962087 10.1038/210204b0

[56] Wilkens LA, Larimer JL 1972 The CNS photoreceptor of crayfish: morphology and synaptic activity. J. Comp. Physiol. 80, 389–407. (doi:10.1007/BF00696436)

[57] Culver DC, Kane T, Fong D 1995 Adaptation and natural selection in caves: the evolution of Gammarus minus. Harvard, MA: Harvard University Press.

[58] Shimizu I, Yamakawa Y, Shimazaki Y, Iwasa T 2001 Molecular cloning of *Bombyx* cerebral opsin (Boceropsin) and cellular localization of its expression in the silkworm brain. Biochem. Biophys. Res. Commun. 287, 27–34. (doi:10.1006/bbrc.2001.5540) 11549248 10.1006/bbrc.2001.5540

[59] Lampel J, Briscoe A, Wasserthal LT 2005 Expression of UV-, blue-, long-wavelength-sensitive opsins and melatonin in extraretinal photoreceptors of the optic lobes of hawkmoths. Cell Tissue Res. 321, 443–458. (doi:10.1007/s00441-004-1069-1) 16034628 10.1007/s00441-004-1069-1

[60] Buschbeck EK, Friedrich M 2008 Evolution of insect eyes: tales of ancient heritage, deconstruction, reconstruction, remodeling and recycling. Evo. Edu. Outreach 1, 448–462. (doi:10.1007/s12052-008-0086-z)

[61] Friedrich M 2008 Development and evolution of the Drosophila Bolwig's organ: a compound eye relict. In Molecular genetics of axial patterning, growth and disease in the Drosophila eye (eds Singh A, Kango-Singh M), pp. 329–357. New York, NY: Springer Science+Business Media.

[62] Langecker TG, Schmale H, Wilkens H 1993 Transcription of the opsin gene in degenerate eyes of cave-dwelling *Astyanax fasciatus* (Teleostei, Characidae) and of its conspecific epigean ancestor during early ontogeny. Cell Tissue Res. 273, 183–192. (doi:10.1007/BF00304625)

[63] Yokoyama R, Yokoyama S 1990 Convergent evolution of the red- and green-like visual pigment genes in fish, *Astyanax fasciatus*, and human. Proc. Natl Acad. Sci. USA 87, 9315–9318. (doi:10.1073/pnas.87.23.9315) 2123554 10.1073/pnas.87.23.9315 PMC55155

