# Supplementary material for: Opsin transcripts of predatory diving beetles: a comparison of surface and subterranean photic niches
Source: R Soc Open Sci. 2015 Jan 28;2(1):140386. doi: 10.1098/rsos.140386 (PMC4448788; doi:10.1098/rsos.140386)
Supplement: ESM for opsins of surface & subterranean diving beetles [file rsos140386supp1.pdf]

SUPPLEMENTARY MATERIAL

Opsin transcripts of predatory diving beetles: a comparison of surface and subterranean photic niches

S.M. Tierney, S.J.B. Cooper, K.M. Saint, T. Bertozzi, J. Hyde, W.F. Humphreys & A.D. Austin

Table of Contents

Table & Figure Legends 2-3
Table S1.1 Blast results for *Allodessus bistrigatus* surface bidessine 4-8
Table S1.2 Blast results for *Limbodessus palmulaoides* subterranean bidessine 9-13
Table S1.3 Blast results for *Neobidessodes gutteridgei* subterranean bidessine 14-18
Table S1.4 Blast results for *Paroster nigroadumbratus* surface hydroporine 19-23
Table S1.5 Blast results for *Paroster macrosturtensis* subterranean bidessine 24-28
Table S2. Orthologue accession numbers 29-30
Table S3. Transcriptome summary 31
Table S4. Tree-branch comparisons of evolutionary rate 32
Figure S1. Workflow diagram 33

TABLE LEGENDS

Table S1. Blast results for the five transcriptome assemblies.

Results are presented in four sub-sections for each species: (1a) amino acid translated queries (BLASTx) of assembled dytiscid beetle contigs to candidate ortholgues (targets), which yields a best hit contig; followed by (1b) reciprocal (tBLASTn) queries; (2) nucleotide match (BLASTn) of best hit dytiscid contigs to the target nucleotide sequence; (3) best Genbank nucleotide match (BLASTn) to the best hit dytiscid contigs; and (4) nucleotide match of one assembled dytiscid contig to another dytiscid species. The latter was only performed when queries 1-3 failed. Abbreviations for associated metrics are as follows: percentage identity (% ID); alignment length (length); query sequence start (qstart); query sequence end (qend); subject sequence start (sstart); subject sequence end (sstart); expect-value measures probability of hit relative to chance alone (evalue); bit score alignment metric (score); homology and length (maxscore); percentage coverage of the query sequence (% Q cover); positive match of query to target (+ve match). Positive matches (≥50 % query coverage & ≥70 % identity) are defined as: Match to target accession (black bar); Match to other insect orthologue (grey bar); or, No evidence for an orthologous match (white bar). An asterisk denotes a discrepancy between the BLASTx and reciprocal tBLASTn best hits.

Table S2. Orthologue accession numbers.

List of orthologous opsin sequences analysed in this study including their opsin class, source organism, gene name, accession number and reference. Full reference citations are included on the following page.

Table S3. Transcriptome sequence and assembly results.

Details the number of raw sequence reads post quality assessment (QA), relative to hardware read length. The number of *de novo* assembled transcripts and assessment of their length, as determined by N50 (50% of assembled contigs that were at least this length); followed by the total number of aligned reads used to generate these assemblies and the proportion of proper-pairs therein. Grey shading indicates a subterranean aphotic niche.

Continued…

Table S4. Variation in evolutionary rates of *long wavelength opsin* among branches.

Likelihood ratio tests on rates of ω (dN/dS) among phylogenetic branches of the (a) dytiscid *lwop* tree, and (b) insect *lwop* tree. For each data matrix, three alternate site-to-site rate variations were assessed: none; dN only; and dN & dS simultaneously. Grey shading indicates a dim-light or aphotic niche. Taxon abbreviations are as follows: *Limbodessus palmulaoides* (Lpal); *Allodessus bistrigatus* (Abis); *Paroster nigroadumbratus* (Pnig); *Thermonectus marmoratus* (Tmar); *Apis mellifera* (Amel); *Papilio xuthus* (Pxut); *Tribolium castaneum* (Tcas); *Bombyx mori* (Bmor); *Maruca vitrata* (Mvit); *Drosophila melanogaster* (Dmel); *Dianemobius nigrofasciatus* (Dnig).

FIGURE LEGEND

Figure S1. Workflow diagram.

Detailed workflow of transcript assembly, orthologous searches based around the Candidate Set of opsin proteins and molecular evolutionary analyses, including phylogenetic reconstruction and tests of selection on longwavength opsin (*lwop*). Major steps are described (black boxes) with associated analytical procedures offset to the right (grey boxes), products derived from these procedures are contained within circles.

Table S1.1.1a Blast results. Surface species A: *Allodessus bistrigatus* BlastX translated AA best contig match.

	Candidate AA	Target name	BlastX Best Hit	% ID	length	qstart	qend	sstart	send	evalue	score	+ve match
	Non-visual opsin											
A1	NP_001035057	Pteropsin [*Apis mellifera*]	comp57640_c0_seq1	47	262	994	1770	47	305	1.00E-67	219	
A2	NP_001138950	Opsin ciliary [*Tribolium castaneum*]	comp57640_c0_seq1	65	284	898	1743	12	295	2.00E-102	315	
	Visual opsin											
A3	BAA93470	UV opsin [*Papilio xuthus*]	comp66921_c0_seq1	57	261	1246	464	25	280	8.00E-108	319	
A4	ABW06837	UV opsin [*Tribolium castaneum*]	comp66921_c0_seq1	74	148	1210	767	1	148	9.00E-82	244	
A5	ACH56537	UV opsin 1 [*Thermonectus marmoratus*]	comp66921_c0_seq1	75	280	1312	473	2	271	2.00E-158	449	
A6	ACH56538	UV opsin 2 [*Thermonectus marmoratus*]	comp66921_c0_seq1	73	280	1303	464	1	271	3.00E-153	435	
A7	NP_001011605	UV opsin [*Apis mellifera*]	comp66921_c0_seq1	65	265	1258	464	10	266	1.00E-126	367	
A8	BAA93469	Blue opsin [*Papilio xuthus*]	comp66921_c0_seq1	43	274	1276	464	8	273	5.00E-83	255	
A9	NP_001011606	Blue opsin [*Apis mellifera*]	comp66921_c0_seq1	43	287	1303	464	1	277	6.00E-79	244	
A10	BAA31721	Rh1 [*Papilio xuthus*]	comp60059_c0_seq1	74	354	251	1312	26	379	0	556	
A11	BAA31722	Rh2 [*Papilio xuthus*]	comp60059_c0_seq1	78	354	251	1312	26	379	0	578	
A12	BAA31723	Rh3 [*Papilio xuthus*]	comp60059_c0_seq1	72	355	248	1312	25	379	0	528	
A13	ACH56536	LW opsin [*Thermonectus marmoratus*]	comp60059_c0_seq1	85	356	248	1312	29	384	0	626	
A14	NP_001011639	LW opsin 1 [*Apis mellifera*]	comp60059_c0_seq1	74	336	251	1258	22	357	0	532	
A15	NP_001071293	LW opsin 2 [*Apis mellifera*]	comp60059_c0_seq1	68	337	248	1258	37	373	8.00E-173	489	
A16	NP_001155991	Rh 1/6 like [*Tribolium castaneum*]	comp60059_c0_seq1	80	355	248	1309	22	376	0	581	

Table S1.1.1b Blast results. Surface species A: *Allodessus bistrigatus* tBlastn - reciprocal blast (transcriptome database).

	Candidate AA	Target name	tBlastn Best Hit	% ID	length	qstart	qend	sstart	send	evalue	score	+ve match
	Non-visual opsin											
A1	NP_001035057	Pteropsin [*Apis mellifera*]	comp57640_c0_seq1	47	266	43	305	982	1770	4.00E-72	241	
A2	NP_001138950	Opsin ciliary [*Tribolium castaneum*]	comp57640_c0_seq1	64	284	12	295	898	1743	5.00E-104	326	
	Visual opsin											
A3	BAA93470	UV opsin [*Papilio xuthus*]	comp66921_c0_seq1	57	261	25	280	1246	464	7.00E-104	318	
A4	ABW06837	UV opsin [*Tribolium castaneum*]	comp66921_c0_seq1	74	148	1	148	1210	767	2.00E-78	244	
A5	ACH56537	UV opsin 1 [*Thermonectus marmoratus*]	comp66921_c0_seq1	75	280	2	271	1312	473	8.00E-155	449	
A6	ACH56538	UV opsin 2 [*Thermonectus marmoratus*]	comp66921_c0_seq1	79	257	1	256	1303	533	6.00E-157	405	
A7	NP_001011605	UV opsin [*Apis mellifera*]	comp66921_c0_seq1	65	265	10	266	1258	464	8.00E-104	318	
A8	BAA93469	Blue opsin [*Papilio xuthus*]	comp66921_c0_seq1	43	274	8	273	1276	464	4.00E-79	254	
A9	NP_001011606	Blue opsin [*Apis mellifera*]	comp66921_c0_seq1	43	287	1	277	1303	464	3.00E-75	244	
A10	BAA31721	Rh1 [*Papilio xuthus*]	comp60059_c0_seq1	74	354	26	379	251	1312	0	568	
A11	BAA31722	Rh2 [*Papilio xuthus*]	comp60059_c0_seq1	78	354	26	379	251	1312	0	593	
A12	BAA31723	Rh3 [*Papilio xuthus*]	comp60059_c0_seq1	72	355	25	379	248	1312	0	540	
A13	ACH56536	LW opsin [*Thermonectus marmoratus*]	comp60059_c0_seq1	85	356	29	384	248	1312	0	636	
A14	NP_001011639	LW opsin 1 [*Apis mellifera*]	comp60059_c0_seq1	74	334	22	355	251	1252	0	532	
A15	NP_001071293	LW opsin 2 [*Apis mellifera*]	comp60059_c0_seq1	68	334	37	379	248	1279	5.00E-165	478	
A16	NP_001155991	Rh 1/6 like [*Tribolium castaneum*]	comp60059_c0_seq1	81	355	22	376	248	1309	0	599	

Table S1.1.2 Blast results. Surface species A: *Allodessus bistrigatus* BlastN – Candidate nucleotide match to contig

	Candidate AA	target name	BlastX Best Hit contig	Candidate nuc seq	max score	% Q cover	% ID	+ve match
	Non-visual opsin							
A1	NP_001035057	Pteropsin [*Apis mellifera*]	comp57640_c0_seq1	no match				
A2	NP_001138950	Opsin ciliary [*Tribolium castaneum*]	comp57640_c0_seq1	no match				
	Visual opsin							
A3	BAA93470	UV opsin [*Papilio xuthus*]	comp66921_c0_seq1	no match				
A4	ABW06837	UV opsin [*Tribolium castaneum*]	comp66921_c0_seq1	no match				
A5	ACH56537	UV opsin 1 [*Thermonectus marmoratus*]	comp66921_c0_seq1	EU164547	174	53	76	
A6	ACH56538	UV opsin 2 [*Thermonectus marmoratus*]	comp66921_c0_seq1	EU921226	437	95	72	
A7	NP_001011605	UV opsin [*Apis mellifera*]	comp66921_c0_seq1	EU921227	499	99	73	
A8	BAA93469	Blue opsin [*Papilio xuthus*]	comp66921_c0_seq1	NM001011605	244	90	68	
A9	NP_001011606	Blue opsin [*Apis mellifera*]	comp66921_c0_seq1	no match	-			
A10	BAA31721	Rh1 [*Papilio xuthus*]	comp60059_c0_seq1	no match	-			
A11	BAA31722	Rh2 [*Papilio xuthus*]	comp60059_c0_seq1	no match	-			
A12	BAA31723	Rh3 [*Papilio xuthus*]	comp60059_c0_seq1	AB007424	446	96	70	
A13	ACH56536	LW opsin [*Thermonectus marmoratus*]	comp60059_c0_seq1	AB007425	379	89	70	
A14	NP_001011639	LW opsin 1 [*Apis mellifera*]	comp60059_c0_seq1	EU921225	735	96	76	
A15	NP_001071293	LW opsin 2 [*Apis mellifera*]	comp60059_c0_seq1	no match				
A16	NP_001155991	Rh 1/6 like [*Tribolium castaneum*]	comp60059_c0_seq1	NM_001162519	500	60	71	

Table S1.1.3 Blast results. Surface species A: *Allodessus bistrigatus* BlastN – best nucleotide match to contig

	Candidate AA	target name	BlastX Best Hit contig	BlastN Best match	max score	% Q cover	% ID	+ve match
	Non-visual opsin							
A1	NP_001035057	Pteropsin [*Apis mellifera*]	comp57640_c0_seq1	XM_004928326	196	95	66	
A2	NP_001138950	Opsin ciliary [*Tribolium castaneum*]	comp57640_c0_seq1	XM_004928326	250	96	67	
	Visual opsin							
A3	BAA93470	UV opsin [*Papilio xuthus*]	comp66921_c0_seq1	EU921227	464	97	74	
A4	ABW06837	UV opsin [*Tribolium castaneum*]	comp66921_c0_seq1	EU921227	291	97	75	
A5	ACH56537	UV opsin 1 [*Thermonectus marmoratus*]	comp66921_c0_seq1	EU921227	500	95	74	
A6	ACH56538	UV opsin 2 [*Thermonectus marmoratus*]	comp66921_c0_seq1	EU921227	499	99	73	
A7	NP_001011605	UV opsin [*Apis mellifera*]	comp66921_c0_seq1	EU921227	464	96	74	
A8	BAA93469	Blue opsin [*Papilio xuthus*]	comp66921_c0_seq1	EU921227	480	99	73	
A9	NP_001011606	Blue opsin [*Apis mellifera*]	comp66921_c0_seq1	EU921227	499	99	73	
A10	BAA31721	Rh1 [*Papilio xuthus*]	comp60059_c0_seq1	EU921227	735	96	76	
A11	BAA31722	Rh2 [*Papilio xuthus*]	comp60059_c0_seq1	EU921225	735	96	76	
A12	BAA31723	Rh3 [*Papilio xuthus*]	comp60059_c0_seq1	EU921225	735	96	76	
A13	ACH56536	LW opsin [*Thermonectus marmoratus*]	comp60059_c0_seq1	EU921225	735	96	76	
A14	NP_001011639	LW opsin 1 [*Apis mellifera*]	comp60059_c0_seq1	EU921225	729	99	76	
A15	NP_001071293	LW opsin 2 [*Apis mellifera*]	comp60059_c0_seq1	EU921225	729	99	76	
A16	NP_001155991	Rh 1/6 like [*Tribolium castaneum*]	comp60059_c0_seq1	EU921225	735	63	76	

Table S1.1.4 Blast results. Surface species A: *Allodessus bistrigatus* BlastN – Blast2Blast contig to another positively matched contig

	Candidate AA	target name	BlastX Best Hit contig	Blast2Blast 2nd contig	max score	% Q cover	% ID	+ve match
	Non-visual opsin							
A1	NP_001035057	Pteropsin [*Apis mellifera*]	comp57640_c0_seq1	comp21388_c0_seq1	682	58	73	
A2	NP_001138950	Opsin ciliary [*Tribolium castaneum*]	comp57640_c0_seq1	comp21388_c0_seq1	682	58	73	
	Visual opsin							
A3	BAA93470	UV opsin [*Papilio xuthus*]	comp66921_c0_seq1	n/a				
A4	ABW06837	UV opsin [*Tribolium castaneum*]	comp66921_c0_seq1	n/a				
A5	ACH56537	UV opsin 1 [*Thermonectus marmoratus*]	comp66921_c0_seq1	n/a				
A6	ACH56538	UV opsin 2 [*Thermonectus marmoratus*]	comp66921_c0_seq1	n/a				
A7	NP_001011605	UV opsin [*Apis mellifera*]	comp66921_c0_seq1	n/a				
A8	BAA93469	Blue opsin [*Papilio xuthus*]	comp66921_c0_seq1	n/a				
A9	NP_001011606	Blue opsin [*Apis mellifera*]	comp66921_c0_seq1	n/a				
A10	BAA31721	Rh1 [*Papilio xuthus*]	comp60059_c0_seq1	n/a				
A11	BAA31722	Rh2 [*Papilio xuthus*]	comp60059_c0_seq1	n/a				
A12	BAA31723	Rh3 [*Papilio xuthus*]	comp60059_c0_seq1	n/a				
A13	ACH56536	LW opsin [*Thermonectus marmoratus*]	comp60059_c0_seq1	n/a				
A14	NP_001011639	LW opsin 1 [*Apis mellifera*]	comp60059_c0_seq1	n/a				
A15	NP_001071293	LW opsin 2 [*Apis mellifera*]	comp60059_c0_seq1	n/a				
A16	NP_001155991	Rh 1/6 like [*Tribolium castaneum*]	comp60059_c0_seq1	n/a				

Table S1.2.1a Blast results. Subterranean species B: *Limbodessus palmulaoides* BlastX - translated AA best contig match

	Candidate AA	target name	BlastX Best Hit	% ID	length	qstart	qend	sstart	send	evalue	score	+ve match
	Non-visual opsin											
B1	NP_001035057	Pteropsin [*Apis mellifera*]	comp29006_c0_seq1	25	327	304	1281	17	327	4.00E-29	108	
B2	NP_001138950	Opsin ciliary [*Tribolium castaneum*]	comp29006_c0_seq1	30	297	322	1209	24	302	8.00E-34	124	
	Visual opsin											
B3	BAA93470	UV opsin [*Papilio xuthus*]	comp29006_c0_seq1	38	344	214	1236	24	365	1.00E-69	223	
B4	ABW06837	UV opsin [*Tribolium castaneum*]	comp29006_c0_seq1	39	152	253	702	1	148	2.00E-26	97	
B5	ACH56537	UV opsin 1 [*Thermonectus marmoratus*]	comp29006_c0_seq1	40	326	253	1215	35	355	5.00E-70	224	
B6	ACH56538	UV opsin 2 [*Thermonectus marmoratus*]	comp29006_c0_seq1	38	333	253	1236	32	359	1.00E-66	214	
B7	NP_001011605	UV opsin [*Apis mellifera*]	comp29006_c0_seq1	42	331	247	1230	24	352	2.00E-77	243	
B8	BAA93469	Blue opsin [*Papilio xuthus*]	comp29006_c0_seq1	36	351	247	1290	31	379	2.00E-64	209	
B9	NP_001011606	Blue opsin [*Apis mellifera*]	comp29006_c0_seq1	36	320	247	1200	33	350	6.00E-64	207	
B10	BAA31721	Rh1 [*Papilio xuthus*]	comp29006_c0_seq1	74	354	229	1290	26	379	0	556	
B11	BAA31722	Rh2 [*Papilio xuthus*]	comp29006_c0_seq1	78	354	229	1290	26	379	0	578	
B12	BAA31723	Rh3 [*Papilio xuthus*]	comp29006_c0_seq1	72	355	226	1290	25	379	0	528	
B13	ACH56536	LW opsin [*Thermonectus marmoratus*]	comp29006_c0_seq1	85	356	226	1290	29	384	0	626	
B14	NP_001011639	LW opsin 1 [*Apis mellifera*]	comp29006_c0_seq1	74	336	229	1236	22	357	0	532	
B15	NP_001071293	LW opsin 2 [*Apis mellifera*]	comp29006_c0_seq1	68	337	226	1236	37	373	1.00E-172	489	
B16	NP_001155991	Rh 1/6 like [*Tribolium castaneum*]	comp29006_c0_seq1	80	355	226	1287	22	376	0	581	

Table S1.2.1b Blast results. Subterranean species B: *Limbodessus palmulaoides* tBlastn - reciprocal blast (transcriptome database)

	Candidate AA	target name	tBlastn Best Hit	% ID	length	qstart	qend	sstart	send	evalue	score	+ve match
	Non-visual opsin											
B1	NP_001035057	Pteropsin [*Apis mellifera*]	comp29006_c0_seq1	26	302	43	328	379	1281	2.00E-24	104	
B2	NP_001138950	Opsin ciliary [*Tribolium castaneum*]	comp29006_c0_seq1	30	297	24	302	322	1209	3.00E-25	107	
	Visual opsin											
B3	BAA93470	UV opsin [*Papilio xuthus*]	comp29006_c0_seq1	38	343	24	364	214	1233	6.00E-66	222	
B4	ABW06837	UV opsin [*Tribolium castaneum*]	comp29006_c0_seq1	39	152	1	148	253	702	2.00E-23	97	
B5	ACH56537	UV opsin 1 [*Thermonectus marmoratus*]	comp29006_c0_seq1	40	326	35	355	253	1215	1.00E-66	224	
B6	ACH56538	UV opsin 2 [*Thermonectus marmoratus*]	comp29006_c0_seq1	38	326	32	352	253	1215	1.00E-53	188	
B7	NP_001011605	UV opsin [*Apis mellifera*]	comp29006_c0_seq1	42	326	24	347	247	1215	4.00E-66	222	
B8	BAA93469	Blue opsin [*Papilio xuthus*]	comp29006_c0_seq1	36	351	31	379	247	1290	2.00E-64	209	
B9	NP_001011606	Blue opsin [*Apis mellifera*]	comp29006_c0_seq1	36	341	33	370	247	1263	3.00E-61	209	
B10	BAA31721	Rh1 [*Papilio xuthus*]	comp29006_c0_seq1	74	354	26	379	229	1290	0	568	
B11	BAA31722	Rh2 [*Papilio xuthus*]	comp29006_c0_seq1	78	354	26	379	229	1290	0	593	
B12	BAA31723	Rh3 [*Papilio xuthus*]	comp29006_c0_seq1	72	355	25	379	226	1290	0	540	
B13	ACH56536	LW opsin [*Thermonectus marmoratus*]	comp29006_c0_seq1	85	356	29	384	226	1290	0	636	
B14	NP_001011639	LW opsin 1 [*Apis mellifera*]	comp29006_c0_seq1	74	334	22	355	229	1230	0	532	
B15	NP_001071293	LW opsin 2 [*Apis mellifera*]	comp29006_c0_seq1	68	334	37	379	226	1257	4.00E-165	478	
B16	NP_001155991	Rh 1/6 like [*Tribolium castaneum*]	comp29006_c0_seq1	81	355	22	376	226	1287	0	599	

Table S1.2.2 Blast results. Subterranean species B: *Limbodessus palmulaoides* BlastN – Candidate nucleotide match to contig

	Candidate AA	target name	BlastX Best Hit contig	Candidate nuc seq	max score	% Q cover	% ID	+ve match
	Non-visual opsin							
B1	NP_001035057	Pteropsin [*Apis mellifera*]	comp29006_c0_seq1	no match				
B2	NP_001138950	Opsin ciliary [*Tribolium castaneum*]	comp29006_c0_seq1	no match				
	Visual opsin							
B3	BAA93470	UV opsin [*Papilio xuthus*]	comp29006_c0_seq1	no match				
B4	ABW06837	UV opsin [*Tribolium castaneum*]	comp29006_c0_seq1	no match				
B5	ACH56537	UV opsin 1 [*Thermonectus marmoratus*]	comp29006_c0_seq1	no match				
B6	ACH56538	UV opsin 2 [*Thermonectus marmoratus*]	comp29006_c0_seq1	no match				
B7	NP_001011605	UV opsin [*Apis mellifera*]	comp29006_c0_seq1	no match				
B8	BAA93469	Blue opsin [*Papilio xuthus*]	comp29006_c0_seq1	no match				
B9	NP_001011606	Blue opsin [*Apis mellifera*]	comp29006_c0_seq1	no match				
B10	BAA31721	Rh1 [*Papilio xuthus*]	comp29006_c0_seq1	AB007423	358	89	69	
B11	BAA31722	Rh2 [*Papilio xuthus*]	comp29006_c0_seq1	AB007424	468	98	70	
B12	BAA31723	Rh3 [*Papilio xuthus*]	comp29006_c0_seq1	AB007425	379	89	70	
B13	ACH56536	LW opsin [*Thermonectus marmoratus*]	comp29006_c0_seq1	EU921225	744	96	76	
B14	NP_001011639	LW opsin 1 [*Apis mellifera*]	comp29006_c0_seq1	no match				
B15	NP_001071293	LW opsin 2 [*Apis mellifera*]	comp29006_c0_seq1	no match				
B16	NP_001155991	Rh 1/6 like [*Tribolium castaneum*]	comp29006_c0_seq1	NM_001162519	459	99	72	

Table S1.2.3 Blast results. Subterranean species B: *Limbodessus palmulaoides* BlastN – Best nucleotide match to contig

	Candidate AA	target name	BlastX Best Hit contig	BlastN Best match	max score	% Q cover	% ID	+ve match
	Non-visual opsin							
B1	NP_001035057	Pteropsin [*Apis mellifera*]	comp29006_c0_seq1	EU921225	690	96	76	
B2	NP_001138950	Opsin ciliary [*Tribolium castaneum*]	comp29006_c0_seq1	EU921225	661	99	77	
	Visual opsin							
B3	BAA93470	UV opsin [*Papilio xuthus*]	comp29006_c0_seq1	EU921225	661	99	77	
B4	ABW06837	UV opsin [*Tribolium castaneum*]	comp29006_c0_seq1	EU921225	738	98	76	
B5	ACH56537	UV opsin 1 [*Thermonectus marmoratus*]	comp29006_c0_seq1	EU921225	306	87	78	
B6	ACH56538	UV opsin 2 [*Thermonectus marmoratus*]	comp29006_c0_seq1	EU921225	717	98	77	
B7	NP_001011605	UV opsin [*Apis mellifera*]	comp29006_c0_seq1	EU921225	724	99	77	
B8	BAA93469	Blue opsin [*Papilio xuthus*]	comp29006_c0_seq1	EU921225	719	98	77	
B9	NP_001011606	Blue opsin [*Apis mellifera*]	comp29006_c0_seq1	EU921225	729	95	77	
B10	BAA31721	Rh1 [*Papilio xuthus*]	comp29006_c0_seq1	EU921225	710	97	77	
B11	BAA31722	Rh2 [*Papilio xuthus*]	comp29006_c0_seq1	EU921225	744	96	76	
B12	BAA31723	Rh3 [*Papilio xuthus*]	comp29006_c0_seq1	EU921225	744	96	76	
B13	ACH56536	LW opsin [*Thermonectus marmoratus*]	comp29006_c0_seq1	EU921225	744	96	76	
B14	NP_001011639	LW opsin 1 [*Apis mellifera*]	comp29006_c0_seq1	EU921225	744	96	76	
B15	NP_001071293	LW opsin 2 [*Apis mellifera*]	comp29006_c0_seq1	EU921225	738	99	76	
B16	NP_001155991	Rh 1/6 like [*Tribolium castaneum*]	comp29006_c0_seq1	EU921225	661	99	77	

Table S1.2.4 Blast results. Subterranean species B: *Limbodessus palmulaoides* BlastN – Blast2Blast contig to another positively matched contig

	Candidate AA	target name	BlastX Best Hit contig	Blast2Blast 2nd contig	max score	% Q cover	% ID	+ve match
	Non-visual opsin							
B1	NP_001035057	Pteropsin [*Apis mellifera*]	comp29006_c0_seq1	n/a				
B2	NP_001138950	Opsin ciliary [*Tribolium castaneum*]	comp29006_c0_seq1	comp57640_c0_seq1	25	1	94	
	Visual opsin							
B3	BAA93470	UV opsin [*Papilio xuthus*]	comp29006_c0_seq1	n/a				
B4	ABW06837	UV opsin [*Tribolium castaneum*]	comp29006_c0_seq1	comp66921_c0_seq1	30	3	75	
B5	ACH56537	UV opsin 1 [*Thermonectus marmoratus*]	comp29006_c0_seq1	n/a				
B6	ACH56538	UV opsin 2 [*Thermonectus marmoratus*]	comp29006_c0_seq1	n/a				
B7	NP_001011605	UV opsin [*Apis mellifera*]	comp29006_c0_seq1	n/a				
B8	BAA93469	Blue opsin [*Papilio xuthus*]	comp29006_c0_seq1	n/a				
B9	NP_001011606	Blue opsin [*Apis mellifera*]	comp29006_c0_seq1	n/a				
B10	BAA31721	Rh1 [*Papilio xuthus*]	comp29006_c0_seq1	n/a				
B11	BAA31722	Rh2 [*Papilio xuthus*]	comp29006_c0_seq1	n/a				
B12	BAA31723	Rh3 [*Papilio xuthus*]	comp29006_c0_seq1	n/a				
B13	ACH56536	LW opsin [*Thermonectus marmoratus*]	comp29006_c0_seq1	n/a				
B14	NP_001011639	LW opsin 1 [*Apis mellifera*]	comp29006_c0_seq1	n/a				
B15	NP_001071293	LW opsin 2 [*Apis mellifera*]	comp29006_c0_seq1	n/a				
B16	NP_001155991	Rh 1/6 like [*Tribolium castaneum*]	comp29006_c0_seq1	comp60059_c0_seq1	2814	97	99	

Table S1.3.1a Blast results. Subterranean species C: *Neobidessoodes gutteridgei* BlastX - translated AA best contig match

	Candidate AA	target name	BlastX Best Hit	% ID	length	qstart	qend	sstart	send	evalue	score	+ve match
	Non-visual opsin											
C1	NP_001035057	Pteropsin [*Apis mellifera*]	comp34192_c0_seq2	24	355	1747	722	8	320	3.00E-18	77	
C2	NP_001138950	Opsin ciliary [*Tribolium castaneum*]	comp34192_c0_seq2	23	336	1690	713	23	337	6.00E-23	94	
	Visual opsin											
C3	BAA93470	UV opsin [*Papilio xuthus*]	comp31049_c0_seq1	24	303	319	1215	62	352	2.00E-22	90	
C4	ABW06837	UV opsin [*Tribolium castaneum*]	comp3041_c0_seq1	33	140	428	841	17	145	2.00E-16	66	
C5	ACH56537	UV opsin 1 [*Thermonectus marmoratus*]	comp31049_c0_seq1	22	288	319	1179	59	337	3.00E-18	77	
C6	ACH56538	UV opsin 2 [*Thermonectus marmoratus*]	comp14164_c0_seq1	25	302	1044	175	51	341	4.00E-16	70	
C7	NP_001011605	UV opsin [*Apis mellifera*]	comp34192_c0_seq2	20	289	1687	845	52	336	1.00E-14	66	
C8	BAA93469	Blue opsin [*Papilio xuthus*]	comp28239_c0_seq2	26	257	954	229	51	297	1.00E-18	77	
C9	NP_001011606	Blue opsin [*Apis mellifera*]	comp34192_c0_seq2	22	283	1669	842	67	345	3.00E-19	81	
C10	BAA31721	Rh1 [*Papilio xuthus*]	comp23676_c0_seq1	31	215	1628	1005	73	279	5.00E-20	82	
C11	BAA31722	Rh2 [*Papilio xuthus*]	comp23676_c0_seq1	23	316	1628	726	73	373	1.00E-16	72	
C12	BAA31723	Rh3 [*Papilio xuthus*]	comp23676_c0_seq1	30	215	1628	1005	73	279	4.00E-18	77	
C13	ACH56536	LW opsin [*Thermonectus marmoratus*]	comp26736_c0_seq1	26	327	1014	55	77	364	7.00E-20	81	
C14	NP_001011639	LW opsin 1 [*Apis mellifera*]	comp23676_c0_seq1	30	215	1628	1005	69	275	4.00E-20	82	
C15	NP_001071293	LW opsin 2 [*Apis mellifera*]	comp23676_c0_seq1	30	206	1628	1032	85	285	3.00E-16	71	
C16	NP_001155991	Rh 1/6 like [*Tribolium castaneum*]	comp28239_c0_seq2	27	257	954	229	49	295	6.00E-18	77	

Table S1.3.1b Blast results. Subterranean species C: *Neobidessoodes gutteridgei* tBlastn - reciprocal blast (transcriptome database)

	Candidate AA	target name	tBlastn Best Hit	% ID	length	qstart	qend	sstart	send	evalue	score	+ve match
	Non-visual opsin											
C1	NP_001035057	Pteropsin [*Apis mellifera*]	comp34192_c0_seq2	24	321	40	320	1284	361	2.00E-13	72	
C2	NP_001138950	Opsin ciliary [*Tribolium castaneum*]	comp34192_c0_seq2	24	317	23	318	1690	770	2.00E-19	91	
	Visual opsin											
C3	BAA93470	UV opsin [*Papilio xuthus*]	comp31049_c0_seq1	24	303	62	352	319	1215	1.00E-18	89	
C4	ABW06837	UV opsin [*Tribolium castaneum*]	comp27425_c0_seq1	32	127	25	148	604	984	1.00E-12	67	
		*BlastX best hit was 2nd	comp3041_c0_seq1	33	140	17	145	428	841	4.00E-13	66	
C5	ACH56537	UV opsin 1 [*Thermonectus marmoratus*]	comp31049_c0_seq1	22	288	59	337	319	1179	8.00E-15	77	
C6	ACH56538	UV opsin 2 [*Thermonectus marmoratus*]	comp14164_c0_seq1	24	302	51	341	1044	175	9.00E-10	61	
C7	NP_001011605	UV opsin [*Apis mellifera*]	comp30557_c0_seq1	25	298	7	299	194	1048	6.00E-10	62	
		*BlastX best hit was 2nd	comp34192_c0_seq2	20	275	65	336	1648	845	1.00E-09	61	
C8	BAA93469	Blue opsin [*Papilio xuthus*]	comp28239_c0_seq2	26	257	51	297	954	229	5.00E-15	77	
C9	NP_001011606	Blue opsin [*Apis mellifera*]	comp14164_c0_seq1	25	319	62	351	1023	139	1.00E-16	82	
		*BlastX best hit was 2nd	comp34192_c0_seq2	22	283	67	345	1669	842	6.00E-16	81	
C10	BAA31721	Rh1 [*Papilio xuthus*]	comp23676_c0_seq1	30	229	59	279	1670	1005	4.00E-21	96	
C11	BAA31722	Rh2 [*Papilio xuthus*]	comp30557_c0_seq1	26	244	46	275	266	970	4.00E-17	84	
		*BlastX best hit was 2nd	comp23676_c0_seq1	25	230	59	279	1670	1005	4.00E-17	84	
C12	BAA31723	Rh3 [*Papilio xuthus*]	comp23676_c0_seq1	30	229	59	279	1670	1005	5.00E-19	90	
C13	ACH56536	LW opsin [*Thermonectus marmoratus*]	comp23676_c0_seq1	26	230	63	283	1670	1005	7.00E-18	86	
		*BlastX best hit was 2nd	comp26736_c0_seq1	26	337	67	364	1044	55	3.00E-17	84	
C14	NP_001011639	LW opsin 1 [*Apis mellifera*]	comp23676_c0_seq1	29	250	37	275	1733	1005	3.00E-23	102	
C15	NP_001071293	LW opsin 2 [*Apis mellifera*]	comp23676_c0_seq1	30	206	85	285	1628	1032	3.00E-16	81	
C16	NP_001155991	Rh 1/6 like [*Tribolium castaneum*]	comp23676_c0_seq1	30	236	52	276	1685	1005	5.00E-20	92	
		*BlastX best hit was 3rd	comp28239_c0_seq2	27	257	49	295	954	229	3.00E-15	77	

Table S1.3.2 Blast results. Subterranean species C: *Neobidessoodes gutteridgei* BlastN – Candidate nucleotide match to contig

	Candidate AA	target name	BlastX Best Hit contig	Candidate nuc seq	max score	% Q cover	% ID	+ve match
	Non-visual opsin							
C1	NP_001035057	Pteropsin [*Apis mellifera*]	comp34192_c0_seq2	no match				
C2	NP_001138950	Opsin ciliary [*Tribolium castaneum*]	comp34192_c0_seq2	no match				
	Visual opsin							
C3	BAA93470	UV opsin [*Papilio xuthus*]	comp31049_c0_seq1	no match				
C4	ABW06837	UV opsin [*Tribolium castaneum*]	comp3041_c0_seq1	no match				
C5	ACH56537	UV opsin 1 [*Thermonectus marmoratus*]	comp31049_c0_seq1	no match				
C6	ACH56538	UV opsin 2 [*Thermonectus marmoratus*]	comp14164_c0_seq1	no match				
C7	NP_001011605	UV opsin [*Apis mellifera*]	comp34192_c0_seq2	no match				
C8	BAA93469	Blue opsin [*Papilio xuthus*]	comp28239_c0_seq2	no match				
C9	NP_001011606	Blue opsin [*Apis mellifera*]	comp34192_c0_seq2	no match				
C10	BAA31721	Rh1 [*Papilio xuthus*]	comp23676_c0_seq1	no match				
C11	BAA31722	Rh2 [*Papilio xuthus*]	comp23676_c0_seq1	no match				
C12	BAA31723	Rh3 [*Papilio xuthus*]	comp23676_c0_seq1	no match				
C13	ACH56536	LW opsin [*Thermonectus marmoratus*]	comp26736_c0_seq1	no match				
C14	NP_001011639	LW opsin 1 [*Apis mellifera*]	comp23676_c0_seq1	no match				
C15	NP_001071293	LW opsin 2 [*Apis mellifera*]	comp23676_c0_seq1	no match				
C16	NP_001155991	Rh 1/6 like [*Tribolium castaneum*]	comp28239_c0_seq2	no match				

Table S1.3.3 Blast results. Subterranean species C: *Neobidessoodes gutteridgei* BlastN – best nucleotide match to contig

	Candidate AA	target name	BlastX Best Hit contig	BlastN Best match	max score	% Q cover	% ID	+ve match
	Non-visual opsin							
C1	NP_001035057	Pteropsin [*Apis mellifera*]	comp34192_c0_seq2	XM_004536657	68	17	70	
C2	NP_001138950	Opsin ciliary [*Tribolium castaneum*]	comp34192_c0_seq2	XM_004536657	68	18	70	
	Visual opsin							
C3	BAA93470	UV opsin [*Papilio xuthus*]	comp31049_c0_seq1	EU138886	307	90	69	
C4	ABW06837	UV opsin [*Tribolium castaneum*]	comp3041_c0_seq1	XM_003400925	237	93	74	
C5	ACH56537	UV opsin 1 [*Thermonectus marmoratus*]	comp31049_c0_seq1	EU138886	306	92	69	
C6	ACH56538	UV opsin 2 [*Thermonectus marmoratus*]	comp14164_c0_seq1	XM_002425220	407	98	71	
C7	NP_001011605	UV opsin [*Apis mellifera*]	comp34192_c0_seq2	XM_004536657	61	20	69	
C8	BAA93469	Blue opsin [*Papilio xuthus*]	comp28239_c0_seq2	XM_005180107	192	48	72	
C9	NP_001011606	Blue opsin [*Apis mellifera*]	comp34192_c0_seq2	XM_004536657	63	21	69	
C10	BAA31721	Rh1 [*Papilio xuthus*]	comp23676_c0_seq1	XM_002056734	304	83	73	
C11	BAA31722	Rh2 [*Papilio xuthus*]	comp23676_c0_seq1	XM_002056734	304	58	73	
C12	BAA31723	Rh3 [*Papilio xuthus*]	comp23676_c0_seq1	XM_002056734	304	83	73	
C13	ACH56536	LW opsin [*Thermonectus marmoratus*]	comp26736_c0_seq1	XM_002429431	123	98	72	
C14	NP_001011639	LW opsin 1 [*Apis mellifera*]	comp23676_c0_seq1	XM_002056734	304	83	73	
C15	NP_001071293	LW opsin 2 [*Apis mellifera*]	comp23676_c0_seq1	XM_002056734	304	87	73	
C16	NP_001155991	Rh 1/6 like [*Tribolium castaneum*]	comp28239_c0_seq2	XM_005180107	192	48	72	

Table S1.3.4 Blast results. Subterranean species C: *Neobidessoodes gutteridgei* BlastN – Blast2Blast contig to another positively matched contig

	Candidate AA	target name	BlastX Best Hit contig	Blast2Blast 2nd contig	max score	% Q cover	% ID	+ve match
	Non-visual opsin							
C1	NP_001035057	Pteropsin [*Apis mellifera*]	comp34192_c0_seq2	n/a				
C2	NP_001138950	Opsin ciliary [*Tribolium castaneum*]	comp34192_c0_seq2	comp57640_c0_seq1	23	2	100	
	Visual opsin							
C3	BAA93470	UV opsin [*Papilio xuthus*]	comp31049_c0_seq1	comp66921_c0_seq1	0	0	0	
C4	ABW06837	UV opsin [*Tribolium castaneum*]	comp3041_c0_seq1	comp66921_c0_seq1	30	1	100	
C5	ACH56537	UV opsin 1 [*Thermonectus marmoratus*]	comp31049_c0_seq1	n/a				
C6	ACH56538	UV opsin 2 [*Thermonectus marmoratus*]	comp14164_c0_seq1	comp66921_c0_seq1	23	0	100	
C7	NP_001011605	UV opsin [*Apis mellifera*]	comp34192_c0_seq2	n/a				
C8	BAA93469	Blue opsin [*Papilio xuthus*]	comp28239_c0_seq2	n/a				
C9	NP_001011606	Blue opsin [*Apis mellifera*]	comp34192_c0_seq2	n/a	25	3	94	
C10	BAA31721	Rh1 [*Papilio xuthus*]	comp23676_c0_seq1	comp60059_c0_seq1	25	0	100	
C11	BAA31722	Rh2 [*Papilio xuthus*]	comp23676_c0_seq1	n/a				
C12	BAA31723	Rh3 [*Papilio xuthus*]	comp23676_c0_seq1	n/a				
C13	ACH56536	LW opsin [*Thermonectus marmoratus*]	comp26736_c0_seq1	comp60059_c0_seq1	23	3	100	
C14	NP_001011639	LW opsin 1 [*Apis mellifera*]	comp23676_c0_seq1	n/a				
C15	NP_001071293	LW opsin 2 [*Apis mellifera*]	comp23676_c0_seq1	n/a				
C16	NP_001155991	Rh 1/6 like [*Tribolium castaneum*]	comp28239_c0_seq2	comp60059_c0_seq1	32	2	100	

Table S1.4.1a Blast results. Surface species D: *Paroster nigroadumbratus* BlastX - translated AA best contig match

	Candidate AA	target name	BlastX Best Hit	% ID	length	qstart	qend	sstart	send	evalue	score	+ve match
	Non-visual opsin											
D1	NP_001035057	Pteropsin [*Apis mellifera*]	comp21388_c0_seq1	48	250	1478	738	47	293	4.00E-69	221	
D2	NP_001138950	Opsin ciliary [*Tribolium castaneum*]	comp21388_c0_seq1	63	289	1583	720	10	298	7.00E-108	325	
	Visual opsin											
D3	BAA93470	UV opsin [*Papilio xuthus*]	comp26389_c3_seq2	61	358	53	1123	7	361	1.00E-165	476	
D4	ABW06837	UV opsin [*Tribolium castaneum*]	comp26389_c3_seq2	72	148	149	592	1	148	2.00E-78	241	
D5	ACH56537	UV opsin 1 [*Thermonectus marmoratus*]	comp26389_c3_seq2	83	358	47	1120	3	357	0	634	
D6	ACH56538	UV opsin 2 [*Thermonectus marmoratus*]	comp26389_c3_seq2	80	357	53	1123	1	355	0	604	
D7	NP_001011605	UV opsin [*Apis mellifera*]	comp26389_c3_seq2	71	335	119	1123	16	350	0	525	
D8	BAA93469	Blue opsin [*Papilio xuthus*]	comp26389_c3_seq2	50	333	125	1123	25	357	5.00E-128	379	
D9	NP_001011606	Blue opsin [*Apis mellifera*]	comp26389_c3_seq2	48	363	53	1123	1	358	1.00E-120	360	
D10	BAA31721	Rh1 [*Papilio xuthus*]	comp18818_c0_seq1	74	334	1257	256	28	361	0	536	
D11	BAA31722	Rh2 [*Papilio xuthus*]	comp18818_c0_seq1	79	334	1257	256	28	361	0	560	
D12	BAA31723	Rh3 [*Papilio xuthus*]	comp18818_c0_seq1	71	334	1257	256	28	361	5.00E-180	506	
D13	ACH56536	LW opsin [*Thermonectus marmoratus*]	comp18818_c0_seq1	86	334	1257	256	32	365	0	610	
D14	NP_001011639	LW opsin 1 [*Apis mellifera*]	comp18818_c0_seq1	74	334	1257	256	24	357	0	526	
D15	NP_001071293	LW opsin 2 [*Apis mellifera*]	comp18818_c0_seq1	70	334	1257	256	40	373	2.00E-174	492	
D16	NP_001155991	Rh 1/6 like [*Tribolium castaneum*]	comp18818_c0_seq1	82	334	1257	256	25	358	0	574	

Table S1.4.1b Blast results. Surface species D: *Paroster nigroadumbratus* tBlastn - reciprocal blast (transcriptome database)

	Candidate AA	target name	tBlastn Best Hit	% ID	length	qstart	qend	sstart	send	evalue	score	+ve match
	Non-visual opsin											
D1	NP_001035057	Pteropsin [*Apis mellifera*]	comp21388_c0_seq1	48	254	43	293	1490	738	5.00E-74	242	
D2	NP_001138950	Opsin ciliary [*Tribolium castaneum*]	comp21388_c0_seq1	63	289	10	298	1583	720	1.00E-109	336	
	Visual opsin											
D3	BAA93470	UV opsin [*Papilio xuthus*]	comp26389_c3_seq2	61	357	7	360	53	1120	3.00E-162	473	
D4	ABW06837	UV opsin [*Tribolium castaneum*]	comp26389_c3_seq2	72	148	1	148	149	592	1.00E-75	241	
D5	ACH56537	UV opsin 1 [*Thermonectus marmoratus*]	comp26389_c3_seq2	83	358	3	357	47	1120	0	633	
D6	ACH56538	UV opsin 2 [*Thermonectus marmoratus*]	comp26389_c3_seq2	80	355	1	353	53	1117	0	556	
D7	NP_001011605	UV opsin [*Apis mellifera*]	comp26389_c3_seq2	71	334	16	349	119	1120	3.00E-161	473	
D8	BAA93469	Blue opsin [*Papilio xuthus*]	comp26389_c3_seq2	50	332	25	356	125	1120	7.00E-125	377	
D9	NP_001011606	Blue opsin [*Apis mellifera*]	comp26389_c3_seq2	48	361	1	356	53	1117	1.00E-116	358	
D10	BAA31721	Rh1 [*Papilio xuthus*]	comp18818_c0_seq1	73	352	28	376	1257	202	0	546	
D11	BAA31722	Rh2 [*Papilio xuthus*]	comp18818_c0_seq1	77	352	28	376	1257	202	0	568	
D12	BAA31723	Rh3 [*Papilio xuthus*]	comp18818_c0_seq1	70	353	28	377	1257	199	0	517	
D13	ACH56536	LW opsin [*Thermonectus marmoratus*]	comp18818_c0_seq1	74	413	3	381	1440	202	0	622	
D14	NP_001011639	LW opsin 1 [*Apis mellifera*]	comp18818_c0_seq1	74	332	24	355	1257	262	0	524	
D15	NP_001071293	LW opsin 2 [*Apis mellifera*]	comp18818_c0_seq1	69	334	40	382	1257	226	3.00E-166	481	
D16	NP_001155991	Rh 1/6 like [*Tribolium castaneum*]	comp18818_c0_seq1	82	352	25	374	1257	256	0	588	

Table S1.4.2 Blast results. Surface species D: *Paroster nigroadumbratus* BlastN – Candidate nucleotide match to contig

	Candidate AA	target name	BlastX Best Hit contig	Candidate nuc seq	max score	% Q cover	% ID	+ve match
	Non-visual opsin							
D1	NP_001035057	Pteropsin [*Apis mellifera*]	comp21388_c0_seq1	no match				
D2	NP_001138950	Opsin ciliary [*Tribolium castaneum*]	comp21388_c0_seq1	no match				
	Visual opsin							
D3	BAA93470	UV opsin [*Papilio xuthus*]	comp26389_c3_seq2	no match				
D4	ABW06837	UV opsin [*Tribolium castaneum*]	comp26389_c3_seq2	EU164547	181	88	76	
D5	ACH56537	UV opsin 1 [*Thermonectus marmoratus*]	comp26389_c3_seq2	EU921226	652	91	75	
D6	ACH56538	UV opsin 2 [*Thermonectus marmoratus*]	comp26389_c3_seq2	EU921227	581	100	72	
D7	NP_001011605	UV opsin [*Apis mellifera*]	comp26389_c3_seq2	NM001011605	307	97	67	
D8	BAA93469	Blue opsin [*Papilio xuthus*]	comp26389_c3_seq2	no match				
D9	NP_001011606	Blue opsin [*Apis mellifera*]	comp26389_c3_seq2	no match				
D10	BAA31721	Rh1 [*Papilio xuthus*]	comp18818_c0_seq1	no match				
D11	BAA31722	Rh2 [*Papilio xuthus*]	comp18818_c0_seq1	no match				
D12	BAA31723	Rh3 [*Papilio xuthus*]	comp18818_c0_seq1	no match				
D13	ACH56536	LW opsin [*Thermonectus marmoratus*]	comp18818_c0_seq1	EU921225	746	99	77	
D14	NP_001011639	LW opsin 1 [*Apis mellifera*]	comp18818_c0_seq1	no match				
D15	NP_001071293	LW opsin 2 [*Apis mellifera*]	comp18818_c0_seq1	no match				
D16	NP_001155991	Rh 1/6 like [*Tribolium castaneum*]	comp18818_c0_seq1	NM_001162519	518	96	72	

Table S1.4.3 Blast results. Surface species D: *Paroster nigroadumbratus* BlastN – best nucleotide match to contig

	Candidate AA	target name	BlastX Best Hit contig	BlastN Best match	max score	% Q cover	% ID	+ve match
	Non-visual opsin							
D1	NP_001035057	Pteropsin [*Apis mellifera*]	comp21388_c0_seq1	XM_004928326	174	53	70	
D2	NP_001138950	Opsin ciliary [*Tribolium castaneum*]	comp21388_c0_seq1	XM_312502	199	91	66	
	Visual opsin							
D3	BAA93470	UV opsin [*Papilio xuthus*]	comp26389_c3_seq2	EU921226	657	91	75	
D4	ABW06837	UV opsin [*Tribolium castaneum*]	comp26389_c3_seq2	EU921226	262	99	73	
D5	ACH56537	UV opsin 1 [*Thermonectus marmoratus*]	comp26389_c3_seq2	EU921226	652	91	75	
D6	ACH56538	UV opsin 2 [*Thermonectus marmoratus*]	comp26389_c3_seq2	EU921226	657	91	75	
D7	NP_001011605	UV opsin [*Apis mellifera*]	comp26389_c3_seq2	EU921226	657	97	75	
D8	BAA93469	Blue opsin [*Papilio xuthus*]	comp26389_c3_seq2	EU921226	657	98	75	
D9	NP_001011606	Blue opsin [*Apis mellifera*]	comp26389_c3_seq2	EU921226	657	91	75	
D10	BAA31721	Rh1 [*Papilio xuthus*]	comp26389_c3_seq2	EU921225	746	99	77	
D11	BAA31722	Rh2 [*Papilio xuthus*]	comp18818_c0_seq1	EU921225	746	99	77	
D12	BAA31723	Rh3 [*Papilio xuthus*]	comp18818_c0_seq1	EU921225	746	99	77	
D13	ACH56536	LW opsin [*Thermonectus marmoratus*]	comp18818_c0_seq1	EU921225	746	99	77	
D14	NP_001011639	LW opsin 1 [*Apis mellifera*]	comp18818_c0_seq1	EU921225	746	99	77	
D15	NP_001071293	LW opsin 2 [*Apis mellifera*]	comp18818_c0_seq1	EU921225	746	99	77	
D16	NP_001155991	Rh 1/6 like [*Tribolium castaneum*]	comp18818_c0_seq1	EU921225	746	99	77	

Table S1.4.4 Blast results. Surface species D: *Paroster nigroadumbratus* BlastN – Blast2Blast contig to another positively matched contig

	Candidate AA	target name	BlastX Best Hit contig	Blast2Blast 2nd contig	max score	% Q cover	% ID	+ve match
	Non-visual opsin							
D1	NP_001035057	Pteropsin [*Apis mellifera*]	comp21388_c0_seq1	n/a				
D2	NP_001138950	Opsin ciliary [*Tribolium castaneum*]	comp21388_c0_seq1	comp57640_c0_seq1	682	58	73	
	Visual opsin							
D3	BAA93470	UV opsin [*Papilio xuthus*]	comp26389_c3_seq2	n/a				
D4	ABW06837	UV opsin [*Tribolium castaneum*]	comp26389_c3_seq2	n/a				
D5	ACH56537	UV opsin 1 [*Thermonectus marmoratus*]	comp26389_c3_seq2	n/a				
D6	ACH56538	UV opsin 2 [*Thermonectus marmoratus*]	comp26389_c3_seq2	n/a				
D7	NP_001011605	UV opsin [*Apis mellifera*]	comp26389_c3_seq2	n/a				
D8	BAA93469	Blue opsin [*Papilio xuthus*]	comp26389_c3_seq2	n/a				
D9	NP_001011606	Blue opsin [*Apis mellifera*]	comp26389_c3_seq2	n/a				
D10	BAA31721	Rh1 [*Papilio xuthus*]	comp18818_c0_seq1	n/a				
D11	BAA31722	Rh2 [*Papilio xuthus*]	comp18818_c0_seq1	n/a				
D12	BAA31723	Rh3 [*Papilio xuthus*]	comp18818_c0_seq1	n/a				
D13	ACH56536	LW opsin [*Thermonectus marmoratus*]	comp18818_c0_seq1	n/a				
D14	NP_001011639	LW opsin 1 [*Apis mellifera*]	comp18818_c0_seq1	n/a				
D15	NP_001071293	LW opsin 2 [*Apis mellifera*]	comp18818_c0_seq1	n/a				
D16	NP_001155991	Rh 1/6 like [*Tribolium castaneum*]	comp18818_c0_seq1	n/a				

Table S1.5.1a Blast results. Subterranean species E: *Paroster macrosturtensis* BlastX - translated AA best contig match

	Candidate AA	target name	BlastX Best Hit	% ID	length	qstart	qend	sstart	send	evalue	score	+ve match
	Non-visual opsin											
D1	NP_001035057	Pteropsin [*Apis mellifera*]	comp26092_c0_seq1	25	313	754	1650	25	314	2.00E-18	77	
D2	NP_001138950	Opsin ciliary [*Tribolium castaneum*]	comp26092_c0_seq2	25	298	146	997	32	308	5.00E-21	86	
	Visual opsin											
D3	BAA93470	UV opsin [*Papilio xuthus*]	comp11778_c0_seq1	23	272	844	56	126	378	1.00E-15	66	
D4	ABW06837	UV opsin [*Tribolium castaneum*]	comp14176_c0_seq1	28	126	542	919	21	145	6.00E-13	58	
D5	ACH56537	UV opsin 1 [*Thermonectus marmoratus*]	comp11778_c0_seq1	23	224	844	182	123	337	2.00E-14	63	
D6	ACH56538	UV opsin 2 [*Thermonectus marmoratus*]	comp26092_c0_seq1	20	290	775	1587	58	341	7.00E-12	57	
D7	NP_001011605	UV opsin [*Apis mellifera*]	comp11778_c0_seq1	22	236	841	158	116	341	5.00E-12	56	
D8	BAA93469	Blue opsin [*Papilio xuthus*]	comp26092_c0_seq1	24	289	766	1587	63	343	7.00E-14	63	
D9	NP_001011606	Blue opsin [*Apis mellifera*]	comp26092_c0_seq1	22	317	772	1674	66	375	6.00E-17	73	
D10	BAA31721	Rh1 [*Papilio xuthus*]	comp6724_c0_seq1	32	128	286	669	73	199	5.00E-16	67	
D11	BAA31722	Rh2 [*Papilio xuthus*]	comp6724_c0_seq1	31	128	286	669	73	199	1.00E-15	66	
D12	BAA31723	Rh3 [*Papilio xuthus*]	comp6724_c0_seq1	31	128	286	669	73	199	1.00E-14	63	
D13	ACH56536	LW opsin [*Thermonectus marmoratus*]	comp26092_c0_seq2	23	309	137	1009	69	369	3.00E-17	73	
D14	NP_001011639	LW opsin 1 [*Apis mellifera*]	comp26092_c0_seq1	23	282	781	1593	64	341	4.00E-16	70	
D15	NP_001071293	LW opsin 2 [*Apis mellifera*]	comp17067_c1_seq1	33	190	647	1195	88	274	2.00E-17	74	
D16	NP_001155991	Rh 1/6 like [*Tribolium castaneum*]	comp5644_c0_seq1	26	282	1792	974	68	345	4.00E-15	70	

Table S1.5.1b Blast results. Subterranean species E: *Paroster macrosturtensis* tBlastn - reciprocal blast (transcriptome database)

	Candidate AA	target name	tBlastn Best Hit	% ID	length	qstart	qend	sstart	send	evalue	score	+ve match
	Non-visual opsin											
D1	NP_001035057	Pteropsin [*Apis mellifera*]	comp26092_c0_seq1	26	274	64	314	740	1522	3.00E-15	77	
D2	NP_001138950	Opsin ciliary [*Tribolium castaneum*]	comp26092_c0_seq2	25	298	32	308	146	997	7.00E-18	84	
	Visual opsin											
D3	BAA93470	UV opsin [*Papilio xuthus*]	comp14176_c0_seq1	25	330	40	348	500	1414	3.00E-15	78	
		*BlastX best hit was 3rd	comp11778_c0_seq1	23	256	126	362	844	104	3.00E-11	64	
D4	ABW06837	UV opsin [*Tribolium castaneum*]	comp14176_c0_seq1	28	126	21	145	542	919	2.00E-11	62	
D5	ACH56537	UV opsin 1 [*Thermonectus marmoratus*]	comp14176_c0_seq1	25	320	36	344	497	1411	1.00E-12	70	
		*BlastX best hit was 2nd	comp11778_c0_seq1	23	224	123	337	844	182	8.00E-11	63	
D6	ACH56538	UV opsin 2 [*Thermonectus marmoratus*]	comp14176_c0_seq1	25	319	33	341	497	1411	6.00E-11	65	
		BlastX best hit was not recovered	comp26092_c0_seq1	20	290	775	1587	58	341	7.00E-12	57	
D7	NP_001011605	UV opsin [*Apis mellifera*]	comp14176_c0_seq1	22	292	65	336	599	1411	4.00E-08	56	
		*BlastX best hit was 2nd	comp11778_c0_seq1	23	236	116	341	841	158	2.00E-08	56	
D8	BAA93469	Blue opsin [*Papilio xuthus*]	comp26092_c0_seq4	24	289	63	343	185	1006	3.00E-13	71	
		*BlastX best hit was 2nd	comp26092_c0_seq1	24	289	63	343	766	1587	5.00E-13	71	
D9	NP_001011606	Blue opsin [*Apis mellifera*]	comp26092_c0_seq1	23	289	66	345	191	1009	8.00E-15	76	
D10	BAA31721	Rh1 [*Papilio xuthus*]	comp24997_c0_seq2	26	249	59	298	1427	732	6.00E-14	74	
		*BlastX best hit was 4th	comp6724_c0_seq1	31	136	61	195	250	657	2.00E-13	70	
D11	BAA31722	Rh2 [*Papilio xuthus*]	comp17067_c1_seq1	27	234	53	282	572	1240	9.00E-15	76	
		*BlastX best hit was 4th	comp6724_c0_seq1	30	140	61	199	250	669	5.00E-13	69	
D12	BAA31723	Rh3 [*Papilio xuthus*]	comp20046_c0_seq1	27	228	68	290	1564	905	9.00E-16	79	
		*BlastX best hit was 6th	comp6724_c0_seq1	32	138	63	199	256	669	1.00E-12	68	
D13	ACH56536	LW opsin [*Thermonectus marmoratus*]	comp17067_c1_seq1	27	231	57	283	572	1240	8.00E-16	79	
		*BlastX best hit was 2nd	comp26092_c0_seq2	23	309	69	369	137	1009	4.00E-15	77	
D14	NP_001011639	LW opsin 1 [*Apis mellifera*]	comp17067_c1_seq1	25	235	49	278	572	1240	2.00E-14	75	
		*BlastX best hit was 4th	comp26092_c0_seq1	23	282	64	341	781	1593	1.00E-12	70	
D15	NP_001071293	LW opsin 2 [*Apis mellifera*]	comp17067_c1_seq1	30	207	88	291	647	1240	3.00E-17	84	
D16	NP_001155991	Rh 1/6 like [*Tribolium castaneum*]	comp24997_c0_seq2	28	249	56	295	1427	732	5.00E-16	80	
		*BlastX best hit was 2nd	comp5644_c0_seq1	26	282	68	345	1792	974	1.00E-12	70	

Table S1.5.2 Blast results. Subterranean species E: *Paroster macrosturtensis* BlastN – Candidate nucleotide match to contig

	Candidate AA	target name	BlastX Best Hit contig	Candidate nuc seq	max score	% Q cover	% ID	+ve match
	Non-visual opsin							
E1	NP_001035057	Pteropsin [*Apis mellifera*]	comp26092_c0_seq1	no match				
E2	NP_001138950	Opsin ciliary [*Tribolium castaneum*]	comp26092_c0_seq2	no match				
	Visual opsin							
E3	BAA93470	UV opsin [*Papilio xuthus*]	comp11778_c0_seq1	no match				
E4	ABW06837	UV opsin [*Tribolium castaneum*]	comp14176_c0_seq1	no match				
E5	ACH56537	UV opsin 1 [*Thermonectus marmoratus*]	comp11778_c0_seq1	no match				
E6	ACH56538	UV opsin 2 [*Thermonectus marmoratus*]	comp26092_c0_seq1	no match				
E7	NP_001011605	UV opsin [*Apis mellifera*]	comp11778_c0_seq1	no match				
E8	BAA93469	Blue opsin [*Papilio xuthus*]	comp26092_c0_seq1	no match				
E9	NP_001011606	Blue opsin [*Apis mellifera*]	comp26092_c0_seq1	no match				
E10	BAA31721	Rh1 [*Papilio xuthus*]	comp6724_c0_seq1	no match				
E11	BAA31722	Rh2 [*Papilio xuthus*]	comp6724_c0_seq1	no match				
E12	BAA31723	Rh3 [*Papilio xuthus*]	comp6724_c0_seq1	no match				
E13	ACH56536	LW opsin [*Thermonectus marmoratus*]	comp26092_c0_seq2	no match				
E14	NP_001011639	LW opsin 1 [*Apis mellifera*]	comp26092_c0_seq1	no match				
E15	NP_001071293	LW opsin 2 [*Apis mellifera*]	comp17067_c1_seq1	no match				
E16	NP_001155991	Rh 1/6 like [*Tribolium castaneum*]	comp5644_c0_seq1	no match				

Table S1.5.3 Blast results. Subterranean species E: *Paroster macrosturtensis* BlastN – best nucleotide match to contig

	Candidate AA	target name	BlastX Best Hit contig	BlastN Best match	max score	% Q cover	% ID	+ve match
	Non-visual opsin							
E1	NP_001035057	Pteropsin [*Apis mellifera*]	comp26092_c0_seq1	XM_003488291	55	8	75	
E2	NP_001138950	Opsin ciliary [*Tribolium castaneum*]	comp26092_c0_seq2	XM_003488291	55	9	75	
	Visual opsin							
E3	BAA93470	UV opsin [*Papilio xuthus*]	comp11778_c0_seq1	EU138886	248	84	69	
E4	ABW06837	UV opsin [*Tribolium castaneum*]	comp14176_c0_seq1	NM_001279451	250	96	75	
E5	ACH56537	UV opsin 1 [*Thermonectus marmoratus*]	comp11778_c0_seq1	EU138886	241	99	68	
E6	ACH56538	UV opsin 2 [*Thermonectus marmoratus*]	comp26092_c0_seq1	XM_003488291	55	9	75	
E7	NP_001011605	UV opsin [*Apis mellifera*]	comp11778_c0_seq1	EU138886	246	97	69	
E8	BAA93469	Blue opsin [*Papilio xuthus*]	comp26092_c0_seq1	XM_003488291	55	9	75	
E9	NP_001011606	Blue opsin [*Apis mellifera*]	comp26092_c0_seq1	XM_003488291	55	8	75	
E10	BAA31721	Rh1 [*Papilio xuthus*]	comp6724_c0_seq1	XM_003401542	295	96	78	
E11	BAA31722	Rh2 [*Papilio xuthus*]	comp6724_c0_seq1	XM_003401542	295	96	78	
E12	BAA31723	Rh3 [*Papilio xuthus*]	comp6724_c0_seq1	XM_003401542	295	96	78	
E13	ACH56536	LW opsin [*Thermonectus marmoratus*]	comp26092_c0_seq2	XM_003488291	55	9	75	
E14	NP_001011639	LW opsin 1 [*Apis mellifera*]	comp26092_c0_seq1	XM_003488291	55	9	75	
E15	NP_001071293	LW opsin 2 [*Apis mellifera*]	comp17067_c1_seq1	AB182633	325	99	74	
E16	NP_001155991	Rh 1/6 like [*Tribolium castaneum*]	comp5644_c0_seq1	XM_005037684	43	3	91	

Table S1.5.4 Blast results. Subterranean species E: *Paroster macrosturtensis* BlastN – Blast2Blast contig to another positively matched contig

	Candidate AA	target name	BlastX Best Hit contig	Blast2Blast 2nd contig	max score	% Q cover	% ID	+ve match
	Non-visual opsin							
E1	NP_001035057	Pteropsin [*Apis mellifera*]	comp26092_c0_seq1	n/a				
E2	NP_001138950	Opsin ciliary [*Tribolium castaneum*]	comp26092_c0_seq2	comp21388_c0_seq1	23	3	100	
	Visual opsin							
E3	BAA93470	UV opsin [*Papilio xuthus*]	comp11778_c0_seq1	comp26389_c3_seq2	23	2	100	
E4	ABW06837	UV opsin [*Tribolium castaneum*]	comp14176_c0_seq1	comp26389_c3_seq2	30	4	100	
E5	ACH56537	UV opsin 1 [*Thermonectus marmoratus*]	comp11778_c0_seq1	n/a				
E6	ACH56538	UV opsin 2 [*Thermonectus marmoratus*]	comp26092_c0_seq1	comp26389_c3_seq2	25	3	94	
E7	NP_001011605	UV opsin [*Apis mellifera*]	comp11778_c0_seq1	n/a				
E8	BAA93469	Blue opsin [*Papilio xuthus*]	comp26092_c0_seq1	n/a				
E9	NP_001011606	Blue opsin [*Apis mellifera*]	comp26092_c0_seq1	n/a				
E10	BAA31721	Rh1 [*Papilio xuthus*]	comp6724_c0_seq1	comp18818_c0_seq1	21	1	93	
E11	BAA31722	Rh2 [*Papilio xuthus*]	comp6724_c0_seq1	n/a				
E12	BAA31723	Rh3 [*Papilio xuthus*]	comp6724_c0_seq1	n/a				
E13	ACH56536	LW opsin [*Thermonectus marmoratus*]	comp26092_c0_seq2	comp18818_c0_seq1	21	0	100	
E14	NP_001011639	LW opsin 1 [*Apis mellifera*]	comp26092_c0_seq1	n/a				
E15	NP_001071293	LW opsin 2 [*Apis mellifera*]	comp17067_c1_seq1	comp18818_c0_seq1	25	3	100	
E16	NP_001155991	Rh 1/6 like [*Tribolium castaneum*]	comp5644_c0_seq1	comp18818_c0_seq1	21	0	100	

Table S2. Orthologue accession numbers

Opsin class & taxon name	Order	Common name	Gene	Accession	Reference
NON-VISUAL OPSIN					
Takifugu rubripes	Tetraodontiformes	Pufferfish	Multiple tissue opsin	AF402774	Moutsaki *et al.* 2003
Danio rerio	Cypriniformes	Zebrafish	Multiple tissue opsin	AF349947	Moutsaki *et al.* 2003
Apis mellifera	Hymenoptera	European honey bee	Pteropsin	NM_001039968	Velarde *et al.* 2005
Tribolium castaneum	Coleoptera	Red flour beetle	C-opsin	NM_001145478	NCBI 2013
Bombyx mori	Lepidoptera	Domestic silkworm	Parapinopsin-like	XM_004928326	NCBI 2013
UV OPSIN					
Papilio xuthus	Lepidoptera	Swallowtail butterfly	UV opsin	AB028218	Kitamoto, *et al.* unpub.
Apis mellifera	Hymenoptera	European honey bee	UV opsin	NM_001011605	Townson *et al.* 1998
Tribolium castaneum	Coleoptera	Red flour beetle	UV opsin-like	XM_965251	NCBI 2008
Thermonectus marmoratus	Coleoptera	Sunburst diving beetle	UV opsin 1	EU921226	Maksimovic *et al.* 2009
Thermonectus marmoratus	Coleoptera	Sunburst diving beetle	UV opsin 2	EU921227	Maksimovic *et al.* 2009
BLUE OPSIN					
Apis mellifera	Hymenoptera	European honey bee	Blue opsin	NM_001011606	Bellingham *et al.* 1997
Papilio xuthus	Lepidoptera	Swallowtail butterfly	Blue opsin	AB028217	Kitamoto, *et al.* unpub.
LONG WAVELENGTH OPSIN					
Tribolium castaneum	Coleoptera	Red flour beetle	Rhodopsin 1/6-like	NM_001162519	Park *et al.* 2008
Thermonectus marmoratus	Coleoptera	Sunburst diving beetle	LW opsin	EU921225	Maksimovic *et al.* 2009
Apis mellifera	Hymenoptera	European honey bee	LW opsin 1	NM_001011639	NCBI 2014
Apis mellifera	Hymenoptera	European honey bee	LW opsin 2	NM_001077825	Velarde *et al.* 2005
Papilo xuthus	Lepidoptera	swallowtail butterfly	Rh1	AB007423	Kitamoto *et al.* 1998
Papilo xuthus	Lepidoptera	swallowtail butterfly	Rh2	AB007424	Kitamoto *et al.* 1998
Papilo xuthus	Lepidoptera	swallowtail butterfly	Rh3	AB007425	Kitamoto *et al.* 1998

REFERENCES (pertaining to Table S2)

Akanuma T. & Nishida H. 2004. Ets-mediated brain induction in embryos of the ascidian *Halocynthia roretzi*. *Development Genes and Evolution* 214: 1-9.

Bellingham J., Wilkie S.E., Morris A.G., Bowmaker J.K. & Hunt D.M. 1997. Characterisation of the ultraviolet-sensitive opsin gene in the honey bee, *Apis mellifera*. *European Journal of Biochemistry* 243: 775-781.

Chang J.-C. & Ramasamy S. 2013. Molecular-Phylogenetic Characterization of Arrestin-2 from *Maruca vitrata* (Lepidoptera: Crambidae). *Annals of the Entomological Society of America* 106: 359-370.

Hoskins R.A. & 13 others. 2007. Sequence finishing and mapping of *Drosophila melanogaster* heterochromatin. *Science* 316: 1625-1628.

Kim H.S. & nine others. 2010. BeetleBase in 2010: revisions to provide comprehensive genomic information for *Tribolium castaneum*. *Nucleic Acids Research* 38: D437-D442.

Kitamoto J., Sakamoto K., Ozaki K., Mishina Y. & Arikawa K. 1998. Two visual pigments in a single photoreceptor cell: identification and histological localization of three mRNAs encoding visual pigment opsins in the retina of the butterfly *Papilio xuthus*. *Journal of Experimental Biology* 201:1255-1261.

Lunt D.H., Zhang D.X., Szymura J.M. & Hewitt G.M. 1996. The insect cytochrome oxidase I gene: evolutionary patterns and conserved primers for phylogenetic studies. *Insect Molecular Biology* 5: 153-165.

Moutsaki P., Whitmore D., Bellingham J., Sakamoto K., David-Gray Z.K. & Foster R.G. 2003. Teleost multiple tissue (tmt) opsin: a candidate photopigment regulating the peripheral clocks of zebrafish? *Molecular Brain Research* 112: 135-145.

Park,Y. & 10 others. 2008. Analysis of transcriptome data in the red flour beetle, *Tribolium castaneum*. *Insect Biochemistry and Molecular Biology* 38: 380-386.

Simon C., Frati F., Beckenbach A., Crespi B., Liu B. & Flook P. 1994. Evolution, weighting and phylogenetic utility of mitochondrial gene sequences and a compilation of conserved polymerase chain reaction primers. *Annals of the Entomological Society of America* 87: 651-701.

Townson S.M., Chang B.S., Salcedo E., Chadwell L.V., Pierce N.E. & Britt S.G. 1998. Honeybee blue- and ultraviolet-sensitive opsins: cloning, heterologous expression in *Drosophila*, and physiological characterization. *Journal of Neuroscience* 18: 2412-2422.

Velarde R.A., Sauer C.D., Walden K.K., Fahrbach S.E. & Robertson H.M. 2005. Pteropsin: a vertebrate-like non-visual opsin expressed in the honey bee brain. *Insect Biochemistry and Molecular Biology* 35: 1367-1377.

Table S3. Transcriptome read and assembly summary.

Taxa	Niche	QA paired-end reads [read length]	*de novo* assembled transcripts [contig N50]	Total aligned reads [% proper pairs]
Bidessini				
Allodessus bistrigatus	surface	78,947,000 [100bp]	182,240 [565 bp]	74,366,408 [58 %]
Limbodessus palmulaoides	subterranean	73,591,991 [100bp]	84,696 [1,067 bp]	55,611,335 [79 %]
Neobidessoides gutteridgei	subterranean	50,857,951 [150bp]	115,296 [904 bp]	37,009,737 [77 %]
Hydroporini				
Paroster nigroadumbratus	surface	75,045,266 [100bp]	84,667 [1,034 bp]	49,105,871 [68 %]
Paroster macrosturtensis	subterranean	50,659,837 [150bp]	76,440 [1,030 bp]	38,738,526 [79 %]

Table S4. Variation in evolutionary rates of *long wavelength opsin* among branches.

site-to-site rate variation	*(a) DYTISCID BEETLES ONLY* Terminal stygal branch *vs* all other branches	Terminal epigean branches *vs* all other branches	All terminal branches *vs* all internal branches
none	X^2_1 = 0.0078, P = 0.930	X^2_3 = 0.2895, P = 0.962	X^2_4 = 0.2975, P = 0.990
Lpal	ω = 0.005 (0-10000)	-	ω = 0.005 (0-10000)
Abis	-	ω = 0 (0-0.09)	ω = 0 (0-0.09)
Pnig	-	ω = 0.005 (0.003-0.007)	ω = 0.005 (0.003-0.007)
Tmar	-	ω = 0.005 (0.004-0.007)	ω = 0.005 (0.004-0.007)
dN only	X^2_1 = -0.0004, P = 1.000	X^2_3 = 4.4071, P = 0.221	X^2_4 = 4.3961, P = 0.355
Lpal	ω = 0.069 (0-10000)	-	ω = 0.068 (0-10000)
Abis	-	ω = 0 (0-0.111)	ω = 0 (0-0.11)
Pnig	-	ω = 0.195 (0.106-0.324)	ω = 0.184 (0.1-0.305)
Tmar	-	ω = 0.274 (0.164-0.429)	ω = 0.259 (0.155-0.405)
dN & dS	X^2_1 = 0.2432, P = 0.622	X^2_3 = 3.6325, P = 0.304	X^2_4 = 3.6719, P = 0.452
Lpal	ω = 0 (0-0.097)	-	ω < 0.001 (0-10000)
Abis	-	ω = 0 (0-0.094)	ω = 0 (0-0.094)
Pnig	-	ω = 0.026 (0.015-0.044)	ω = 0.027 (0.015-0.045)
Tmar	-	ω < 0.001 (0.0001-0.0003)	ω < 0.001 (0 - 0.0002)

	(b) OTHER INSECTS Terminal stygal branch *vs* all other branches	Terminal epigean branches *vs* all other branches	All terminal branches *vs* all internal branches
none	X^2_1 = 0.182513, P = 0.669	X^2_3 = 4.19217, P = 0.241	X^2_{10} = 15.1426, P = 0.127
Lpal	ω = 0 (0 - 0.314)	-	ω = 0.035 (0-10000)
Abis	-	ω = 0 (0 - 0.102)	ω = 0 (0-0.102)
Pnig	-	ω = 0.037 (0.024-0.052)	ω = 0.035 (0.023-0.050)
Tmar	-	ω = 0.116 (0.069-0.179)	ω = 0.101 (0.060-0.156)
Amel1	-	-	ω = 0.008 (0.006-0.011)
Amel2	-	-	ω = 0.012 (0.009-0.014)
Pxut1	-	-	ω = 0.025 (0.014-0.041)
Pxut2	-	-	ω = 0.066 (0.045-0.091)
Pxut3	-	-	ω = 0.034 (0.015-0.061)
Tcas	-	-	ω = 0.064 (0.044-0.088)
dN only	X^2_1 = 0.0883136, P = 0.766	X^2_3 = 3.91313, P = 0.271	X^2_{10} = 10.7563, P = 0.377
Lpal	ω = 0 (0-0.454)	-	ω = 0.047 (0-10000)
Abis	-	ω = 0 (0-0.102)	ω = 0 (0-0.103)
Pnig	-	ω = 0.039 (0.02-0.069)	ω = 0.038 (0.023-0.057)
Tmar	-	ω = 0.128 (0.069-0.212)	ω = 0.111 (0.059-0.184)
Amel1	-	-	ω = 0.016 (0.011-0.024)
Amel2	-	-	ω = 0.027 (0.02-0.036)
Pxut1	-	-	ω = 0.0252 (0.013-0.043)
Pxut2	-	-	ω = 0.031 (0.011-0.063)
Pxut3	-	-	ω = 0.071 (0.047-0.102)
Tcas	-	-	ω = 0.08 (0.053-0.116)
dN & dS	X^2_1 = 0.0121743, P = 0.912	X^2_3 = 3.55792, P = 0.313	X^2_{10} = 13.848, P = 0.18
Lpal	ω = 0.011 (0-10000)	-	ω = 0.009 (0-10000)
Abis	-	ω = 0 (0-0.099)	ω = 0 (0-0.097)
Pnig	-	ω = 0.008 (0.004-0.013)	ω = 0.006 (0.004-0.008)
Tmar	-	ω = 0.108 (0.056-0.182)	ω = 0.183 (0.095-0.308)
Amel1	-	-	ω = 0.002 (0.001-0.003)
Amel2	-	-	ω = 0.001 (0.001-0.002)
Pxut1	-	-	ω = 0.006 (0.003-0.009)
Pxut2	-	-	ω = 0.033 (0.011-0.068)
Pxut3	-	-	ω = 0.012 (0.008-0.018)
Tcas	-	-	ω = 0.021 (0.014-0.031)

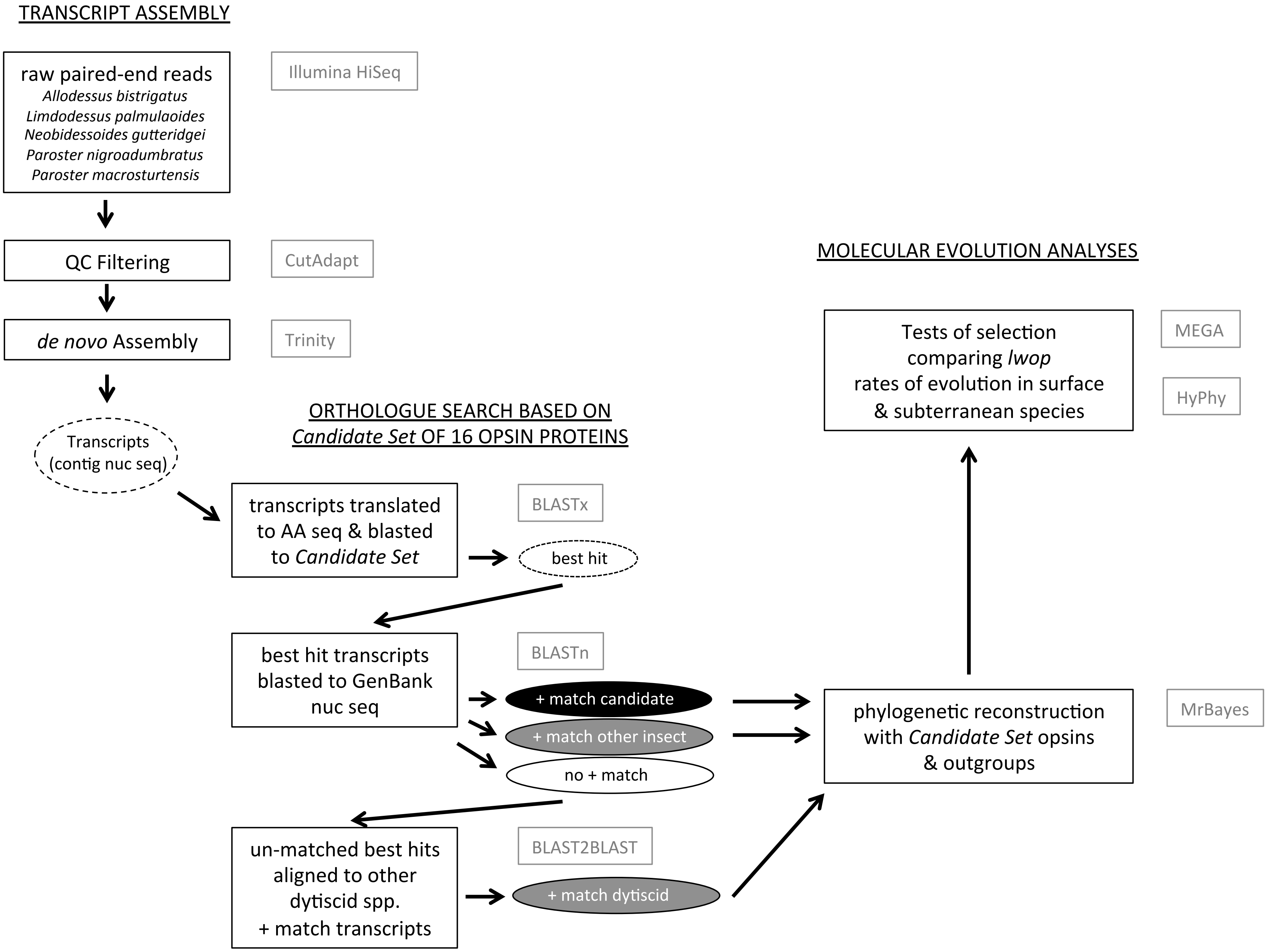
TRANSCRIPT ASSEMBLY
raw paired-end reads
Allodessus bistrigatus
Limdodessus palmulaoides
Neobidessoides gutteridgei
Paroster nigroadumbratus
Paroster macrosturtensis
Illumina HiSeq
QC Filtering
CutAdapt
de novo Assembly
Trinity
Transcripts (contig nuc seq)
ORTHOLOGUE SEARCH BASED ON Candidate Set OF 16 OPSIN PROTEINS
transcripts translated to AA seq & blasted to Candidate Set
BLASTx
best hit
best hit transcripts blasted to GenBank nuc seq
BLASTn
+ match candidate
+ match other insect
no + match
un-matched best hits aligned to other dytiscid spp. + match transcripts
BLAST2BLAST
+ match dytiscid
MOLECULAR EVOLUTION ANALYSES
Tests of selection comparing lwop rates of evolution in surface & subterranean species
MEGA
HyPhy
phylogenetic reconstruction with Candidate Set opsins & outgroups
MrBayes
